# Quantified electrostatic preorganization in enzymes using the geometry of the electron charge density†
†Electronic supplementary information (ESI) available: ESI includes a discussion of QTAIM, table of amino acid residues in each size model, topological connections in the central region, plots of charge density analysis results using a variety of functionals and basis sets, plots of atomic volumes, and nuclear coordinates for simulations. See DOI: 10.1039/c7sc01301a



**DOI:** 10.1039/c7sc01301a

**Published:** 2017-04-24

**Authors:** Amanda Morgenstern, Matthew Jaszai, Mark E. Eberhart, Anastassia N. Alexandrova

**Affiliations:** a Molecular Theory Group , Colorado School of Mines , USA . Email: meberhar@mines.edu; b Department of Chemistry and Biochemistry , University of California , Los Angeles , USA . Email: ana@chem.ucla.edu

## Abstract

Electrostatic preorganization is thought to be a principle factor responsible for the impressive catalytic capabilities of enzymes. The full protein structure is believed to facilitate catalysis by exerting a highly specific electrostatic field on the active site. Computationally determining the extent of electrostatic preorganization is a challenging process. We propose using the topology and geometry of the electron charge density in the enzyme's active site to asses the effects of electrostatic preorganization. In support of this approach we study the convergence of features of the charge density as the size of the active site model increases in Histone Deacetylase 8. The magnitude of charge density at critical points and most Bader atomic charges are found to converge quickly as more of the protein is included in the simulation. The exact position of critical points however, is found to converge more slowly and be strongly influenced by the protein residues that are further away from the active site. We conjecture that the positions of critical points are affected through perturbations to the wavefunctions in the active site caused by dipole moments from amino acid residues throughout the protein. We further hypothesize that electrostatic preorganization, from the point of view of charge density, can not be easily understood through the charges on atoms or the nature of the bonding interactions, but through the relative positions of critical points that are known to correlate with reactivity and reaction barriers.

## Abstract 2

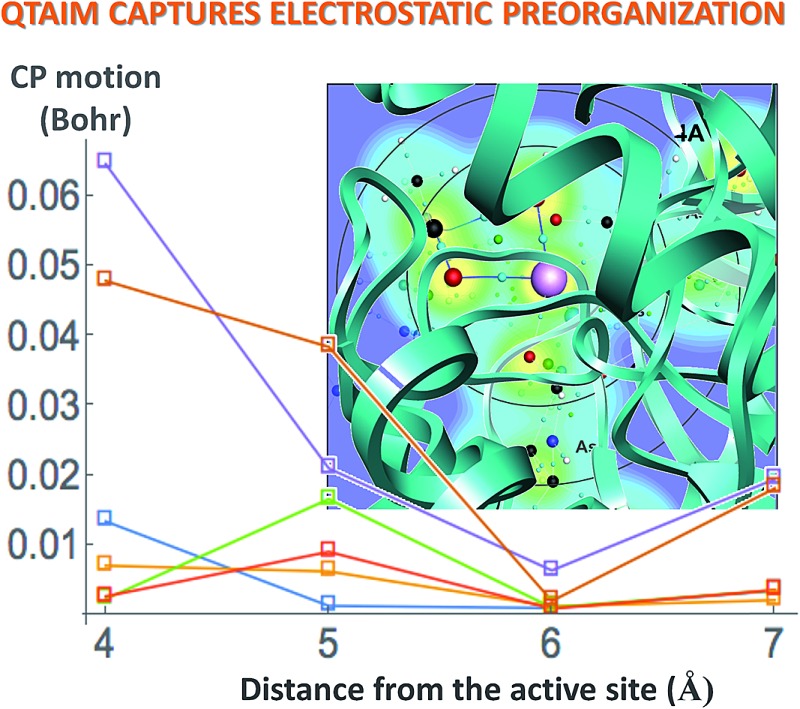
The exact positions of critical points in the charge density in enzyme active sites reflects electrostatic preorganization.

## 1 Introduction

Understanding enzymatic function, including the exact mechanism, and the factors making these catalysts efficient and incredibly selective, is central to chemistry, biology, and medicine. It is also important in industrial catalysis if we wish to design equally efficient artificial enzymes for unnatural functions of interest to humankind.^1,2^ The field has advanced tremendously over the years, including successes in artificial enzyme design, but there is one aspect that is still suspended in the state of a hypothesis. While only a handful of amino acids in the active site participate in the reaction, the rest of the protein macromolecule is proposed to have an important function too, and that is the creation of a highly specific electric field. That field would influence the active site and bound substrate, and interact most beneficially with the transition state of the catalyzed reaction.^3^

This phenomenon is known as electrostatic preorganization, and has been argued to be the origin of the enormous catalytic power of enzymes.^3,4^ The full enzyme, particularly the polar groups, are thought to perturb the electron density at the active site to create an electrostatic field that lowers the activation barrier to the catalytic reaction. Electrostatic interactions are long-ranged, and this gives insight into the need for the large size of enzymes. When a reaction proceeds in a polar solution such as water, the solute molecules see a fluctuating dipole environment caused by the motion of the solvent molecules. For a great many catalytic reactions, the transition state energy is highly sensitive to this environment, with the lowest transition state energy characterized by a distinct organization of the solvent dipoles. The free energy difference between the reactant and transition states is what determines the rate constant in enzyme catalysis, making the transition state free energy a key component to optimize for reaction speed. It is suggested that the charged and polar residues – even at some distance from the active site – are positioned in enzymes so as to permanently preorganize the electric field and thereby minimize the free energy of activation.

While electrostatic preorganization is gaining acceptance as the largest factor in catalytic ability of enzymes,^5,6^ assessing its extent remains a challenge. Some computational and experimental methods have been used to determine the role of electrostatic preorganization and quantify its influence on activation barriers. Computationally, empirical valence bond (EVB) methods have been used to determine the importance of electrostatic interactions based on contributions from ionic resonance forms along the reaction coordinate. The stabilization of the ionic resonance form at the transition state has been found to be linearly related to the difference in activation free energies for an enzymatic reaction occurring in a general solvent compared to the enzyme.^5–7^ A similar relationship is not found for the covalent resonance forms, suggesting that electrostatic stabilization of ionic structures is a key feature for enzymatic reactions. Experimentally, vibrational Stark spectroscopy has been used to measure local dipole moments in the active site that are influenced by an external field. Direct relationships between dipole moments in carbonyl and nitrile functional groups to activation energies for enzyme catalyzed reactions have been found.^8–11^

**Fig. 1:**
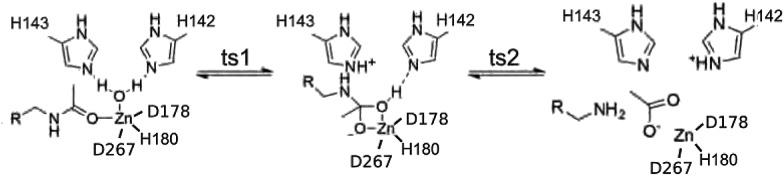
Proton shuttle mechanism for HDAC8.

We take a novel approach to investigate electrostatic preorganization by following the evolution of the electron charge density, *ρ*, due to successive perturbations to the environment around an enzyme active site. The electrostatic potential is determined by the electron charge density *via* the Poisson equation, making *ρ* a logical platform for studying electrostatics in enzymes. Furthermore, the active site is electronically polarizable, so certain structural features in the electron density will change when a polar environment, *i.e.* the full protein, is added around the active site. We use Histone Deacetylase 8 (HDAC8), which catalyzes a deacetylation reaction, as our model. HDAC8 is a well-studied enzyme for which a variety of mechanisms have been proposed,^12–15^ but we have found the proton shuttle mechanism presented in Fig. 1 to be the most viable.^16^ Through this mechanism, H143 acts first as a base, abstracting a proton from the water molecule, and second as an acid, transferring the proton to the substrate.

**Fig. 2:**
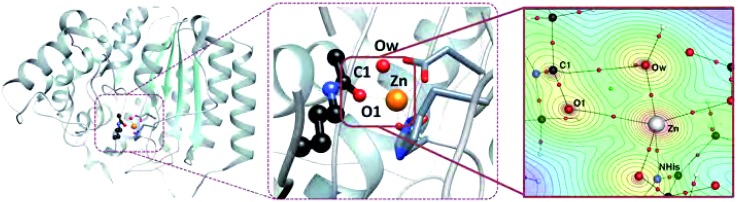
Left: Full HDAC8 structure showing position of active site. Center: Active site of HDAC8 with zinc ion, water molecule, and part of the substrate molecule shown in ball and stick model. Right: Topological structure of HDAC8 enzyme–substrate complex from nuclear configuration 1 zoomed in on atoms of interest. A contour plot of *ρ* is shown through the plane defined by the Zn^2+^, Ow, and O1 atoms. Bond paths are shown with black lines, bond CPs are small red spheres, and ring CPs are small green spheres. Atoms involved in key bonding interactions for enzyme function and metal binding are labelled.

Key bonding interactions for this reaction were found to involve the substrate carbonyl (C1 and O1), the oxygen on the water molecule (Ow) and the Zn^2+^ ion (see Fig. 2). These four atoms form the tetrahedral intermediate after ts1. Furthermore, the amount of charge density at the bond critical point (CP), a saddle point in *ρ*, between the zinc ion and a nitrogen atom in H180 (NHis) was found to be linearly correlated to metal binding affinity for HDAC8.^16^ In this study, we thus focus on the charge density around these five atoms, which are highlighted in Fig. 2.

Our approach is as follows: for three different nuclear configurations obtained from quantum mechanics/discrete molecular dynamics (QM/DMD) simulations we approximate the enzymatic structure around the divalent zinc ion with five essentially spherical clusters of increasing radii. We then use density functional methods to compute the one electron wave functions and charge density for each of the 15 resulting model systems. By analyzing the changes to the density through a series of increasingly larger clusters, we are able to separate the enzyme's atoms into those that are part of a local environment that directly contributes charge density to the region under study, consisting of the Zn^2+^, C1, O1, Ow, and NHis atoms (henceforth referred to as the central region), and those that do not. The latter region is an extended environment whose effect on the deacetylation reaction is to perturb the electronic structure of the reactive region. The magnitude of the perturbation can be assessed by the induced changes to the charge density in the central region which are mirrored by changes to the corresponding CPs in this region.

Our paper is organized by first determining the boundary of the local environment in the active site of HDAC8. We find this boundary by measuring where Bader atomic charges and the amount of charge density at CPs in the central region converge due to direct overlap of one-electron wave functions. Next we discuss the implications of perturbation theory on the CP structure. We study the perturbative effect of the extended environment on the central region by measuring the exact positions of CPs in the central region, which we propose can be used as an indicator of electrostatic preorganization. Then we discuss the importance of the potassium ion near the HDAC8 active site on the motion of CPs. Finally, a discussion of results and their implications for enzyme design is presented.

## 2 Computational methods and theory

In order to analyze the effects of the protein's extended environment on electrostatic preorganization we must first determine the position of the boundary surrounding the local environment, outside of which all other atoms will act as a perturbation on the central region charge density. The boundary is found by moving radially out from the zinc ion and determining when additional atomic wavefunctions no longer contribute significant charge density to the central region. We use two methods based on the topology of the electron charge density to measure when the magnitude of *ρ* in the central region has converged. First we measure the Bader atomic charges of the Zn^2+^, C1, O1, Ow, and NHis atoms. Next we find the size of the active site cluster model for which the magnitude of charge density at bond and ring CPs in the central region converges. For details on how Bader charges and CPs are defined and calculated, see ESI.†

Nuclear coordinates from the three lowest energy configurations obtained from QM/DMD simulations performed by Nechay *et al.* were used to create our active site models for these calculations (see Table S1 and Fig. S1 in ESI†).^16^ Details on QM/DMD can be found elsewhere.^17^ The largest active site models were determined by including any residue with at least one atom within 7 Å of the Zn^2+^ ion. Capping hydrogens were added to amino acids where necessary and their geometries were optimized, freezing the coordinates of the rest of the model. Smaller models (3–6 Å) were created by removing any amino acid residues from the 7 Å models that did not include atoms within the cut-off distance and again optimizing the positions of capping hydrogens. Single point calculations were performed on systems of all five sizes for the three configurations using all combinations of double-zeta polarized (DZP) and triple-zeta polarized (TZP) Slater-type orbital basis sets with four functionals: LDA, PBE, M06L, and TPSS, within the Amsterdam Density Functional (ADF) package version 2016.^18–21^ This resulted in a total of 120 single point calculations. The topology and geometry of the electron charge density was analyzed using the standard Bader package within ADF^22,23^ to calculate Bader atomic charges and magnitude of charge density at CPs.

Calculations larger than 7 Å were not computationally feasible due to system sizes exceeding 600 atoms. We instead approximated the effects of the protein beyond our largest cluster model using two methods for the 7 Å LDA TZP configuration 1 active site model. The first approximation method was to add a dielectric constant to the calculation using COSMO solvation with *ε* = 4 and 20 to represent the full protein, which are common values used in enzyme calculations for buried and surface active sites, respectively. The second approach was to add point charges from the surrounding protein environment to the single point calculations. Point charges were obtained using NWChem^24^ and were calculated using a Co^2+^ HDAC8 system from ref. 16. The point charge calculation is expensive, so we chose not to repeat the calculation for the zinc HDAC8 system since the RMSD between the full protein of the Co^2+^ and Zn^2+^ systems was nearly zero. The electrostatic embedding calculation performed in ADF included 3000 point charges calculated from atoms within a 25 Å radius of the Zn^2+^ ion.

## 3 Results

### 3.1 Atomic charges

Bader atomic charges for Zn^2+^, C1, O1, Ow, and NHis from the single point LDA TZP calculations are shown in Fig. 4. The three lines for each color are representative of the three different starting nuclear coordinate configurations. Table S1 (ESI†) lists the amino acids included in each model size. Despite containing different atoms in most model sizes, configuration 1 yields similar results as the other two configurations. Even when moving from the 5–6 Å model size, where configuration 1 adds four amino acids and configurations 2 and 3 only add one amino acid, changes in atomic charges have the same trends between the three configurations. Though their exact geometries differ, configurations 2 and 3 have the same amino acid residues in each size model except that configuration 3 does not include the potassium ion in any size model.

**Fig. 3:**
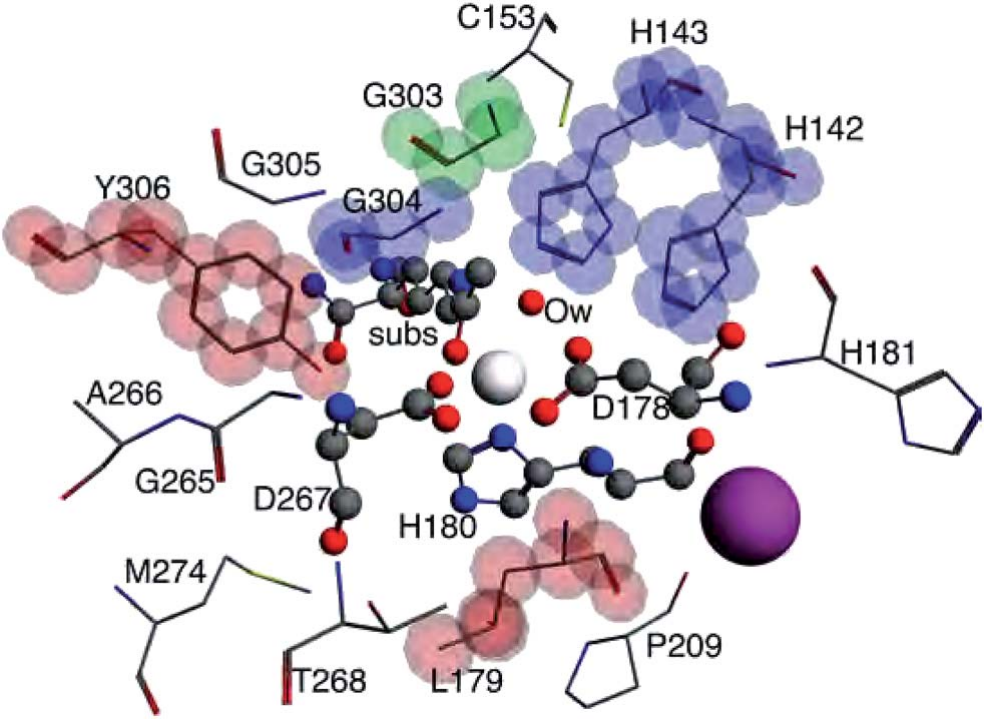
The 3–7 Å size systems for HDAC8 from nuclear configuration 2. The 3 Å system consists of the central Zn^2+^ ion (white sphere), substrate (subs), water molecule (Ow), D178, H180, and D267, all shown in ball and stick model. The 4 Å system adds L179 and Y306, shown with stick model highlighted in red. 5 Å adds H142, H143, and G304, stick model highlighted in blue. 6 Å includes G303, highlighted in green. The largest 7 Å system includes a K^+^ ion (purple sphere) and C153, H181, P209, G265, A266, T268, M274, and G305, shown with non-highlighted stick model.

**Fig. 4:**
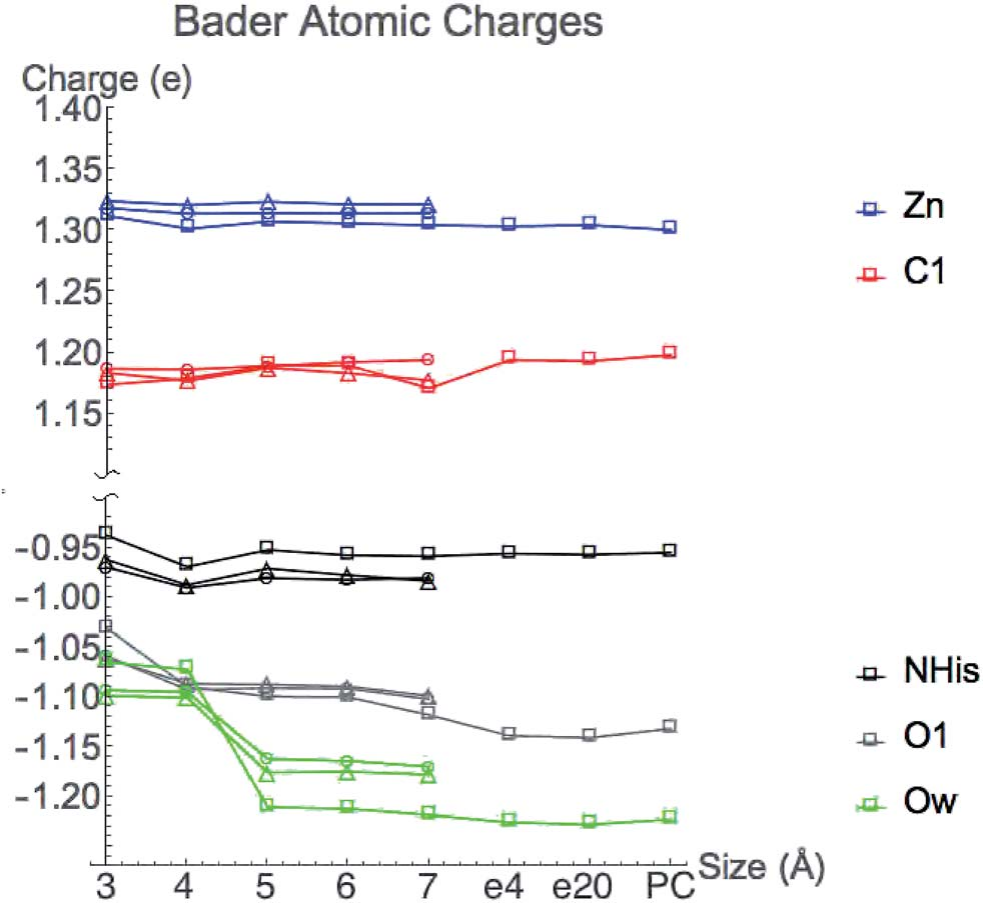
Bader atomic charges from TZP LDA calculations as active site model sizes increase from 3–7 Å. The three lines for each atom represent the three different starting QM/DMD nuclear configurations with the following shapes: configuration 1-square, 2-circle, 3-triangle. Configuration 1 also includes points for 7 Å system with dielectric constants of 4 (e4) and 20 (e20) and with point charges (PC).

Most Bader atomic charges for HDAC8 converge once a full residue sphere is included around each atom in the central region. The O1 charge initially appears to converge at 4 Å, once Tyr306 is included (which hydrogen bonds to O1), with the most pronounced change occurring for configuration 1. However, from 6–7 Å the O1 charge decreases slightly, most notably for nuclear configuration 1. The Ow charge does not converge until the model sizes reaches 5 Å, which includes the His142 and 143 that hydrogen bond to the water molecule (see Table S1 (ESI†) and Fig. 3). The charge on NHis initially decreases at 4 Å, but increases to its original 3 Å charge value after this point. All other atomic charges remain fairly constant starting from the base level 3 Å model. These results are consistent across all functionals and basis sets tested as shown in Fig. S2 (ESI†).

There is some variation in atomic charges beyond the 7 Å system for configuration 1 from COSMO solvation and point charge calculations. The largest difference is the O1 charge using COSMO solvation with *ε* = 20 (0.015 electrons), which is also the atom with the least converged charge for the 7 Å cluster model. The C1 charge increases when adding a dielectric constant or point charges, but still remains in the same range of values from the 5–6 Å model calculations for configuration 1 without the full protein effect added. Since the C1 charge changes in the opposite direction of O1, this indicates that the overall substrate carbonyl charge is remaining constant, but the group gets polarized and activated. In general, inverse behavior was found for atomic volumes, *i.e.* an increase in atomic charge (decrease in number of electrons) correlates to a decrease in atomic basin volumes (Fig. S3 (ESI†)), as has been found in previous studies.^25^ All other atoms have negligible change in their converged values from the addition of point charges or COSMO solvation.

Atomic charges are generally converged when one residue sphere is included around the atom of interest, but have slight variations for some values at larger sized cluster models. Bader atomic charges are based not only on the magnitude of *ρ*, but also on the locations of their boundaries that are influenced by the exact position of CPs. As will be discussed in Section 3.3, Bader charges may also be affected by perturbations from the extended environment and their convergence rate may not be the best metric to determine the boundary of the local environment. Interestingly, previous work showed that Bader atomic charges in the active site of 2-cysteine peroxiredoxin thioredoxin peroxidase B converge rapidly for neutral atoms as the model size increases, but not for negatively charged atoms.^26^ Here we have tested one positively charged atom, Zn^2+^, and found its charge to rapidly converge.

### 3.2 Magnitude of *ρ* at critical points

**Fig. 5:**
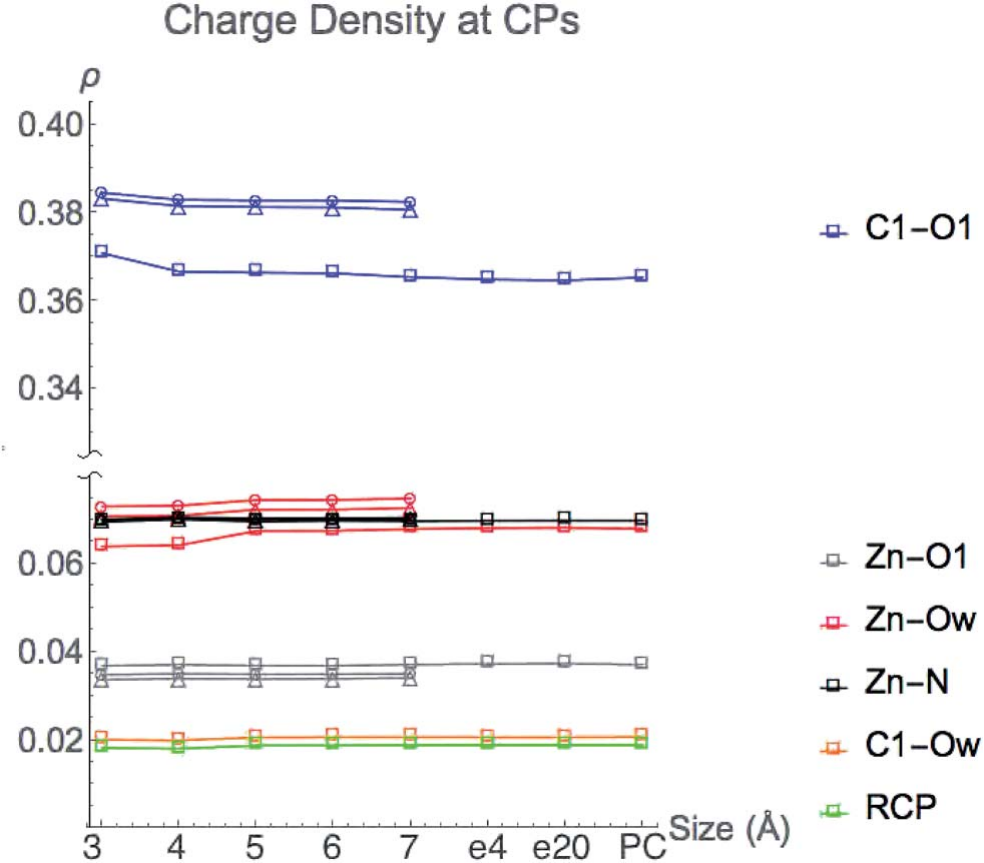
Charge density at critical points from LDA TZP calculations as active site model sizes increase in size (3–7 Å). The three lines for each atom represent the three different starting QM/DMD configurations used with the following shapes: configuration 1-square, 2-circle, 3-triangle. The C1–Ow bond CP and the ring CP are only present for configuration 1. Configuration 1 also includes points for 7 Å system with dielectric constants of 4 (e4) and 20 (e20) and with point charges (PC).

The value of charge density at bond and ring critical points in the central region of HDAC8 from single point LDA TZP calculations are displayed in Fig. 5. The three nuclear configurations yield similar results for the magnitude of *ρ* at CPs with the exceptions of the C1–O1 and Zn^2+^–Ow configuration 1 values. These critical points are one bond path away from the incipient C1–Ow bond CP, a result of the close proximity to the ring CP. These two CPs can annihilate each other as a result of slight nuclear motions, as occurs in nuclear configurations 2 and 3 (see Fig. S1 (ESI†)). In configuration 1, the C1–Ow bond path enables some of the charge density to be pulled out of both the carbonyl bonding region and the Zn^2+^–Ow interaction, which for configuration 1 results in lowered values of *ρ* at these CPs. Even though the values of *ρ* vary at these two points for configuration 1, the trends of the variation in *ρ* as the model size increases are consistent with configurations 2 and 3.

Once all direct bonding interactions to the central region are included in the model size, the CP charge density converges. The only critical points where a system larger than the 3 Å base model is required for converged values of *ρ* are the C1–O1 and Zn^2+^–Ow bond CPs. The magnitude of *ρ* at the Zn^2+^–Ow bond CP is converged for the 5 Å system once His142 and His143 are included. The C1–O1 bond CP reaches a converged value of *ρ* for the 4 Å model size, once Tyr306 is included. Configurations 2 and 3 have a slight depression in the amount of *ρ* at the C1–O1 bond CP from 3–4 Å but there is a larger change in configuration 1. The differences in the change in *ρ* are likely due to the Tyr306 hydrogen bonding occurring with an oxygen atom in configuration 1, and a carbon atom on Tyr306 in configurations 2 and 3.

Adding either point charges or a dielectric constant to the active site cluster model calculation does not affect the magnitude of *ρ* at CPs. The converged values that are reached by the 5 Å model remain constant when approximating electrostatic effects from the full protein, emphasizing that the magnitude of *ρ* at CPs is due to the local environment around the central region.

We conclude that the boundary of the local environment occurs when one residue sphere has been added around each CP in the central region. The magnitude of *ρ* is most affected by direct changes in *ρ* due to an overlap of wavefunctions in the base system with new atomic wavefunctions that are introduced from directly neighboring amino acids. Since the charge density drops off exponentially, only nearby molecular orbitals have large effects on the magnitude of *ρ*. Once a full residue sphere is included around a critical point in the central region, the magnitude of *ρ* at the point is converged. Results from other functionals and basis sets give similar results and are shown in Fig. S4 (ESI†).

### 3.3 Critical point position

Since the changes to the charge density in the central region are subtle, we use a perturbative approach to examine the effect of the extended environment of the protein. We conjecture that the perturbation from the full protein can be observed through the position of CPs that are defined by points where the gradient of the charge density, ∇*ρ*, vanishes. Consider each successive sphere added to the base model of HDAC8 as a perturbation to the Hamiltonian, which acts on the molecular orbitals in the central region. This perturbation from the full enzyme environment can be represented by a multipole expansion centered on the zinc ion. The first order corrections to the unperturbed one-electron wavefunctions in the central region, *ψ*(0)*j*, are given by1
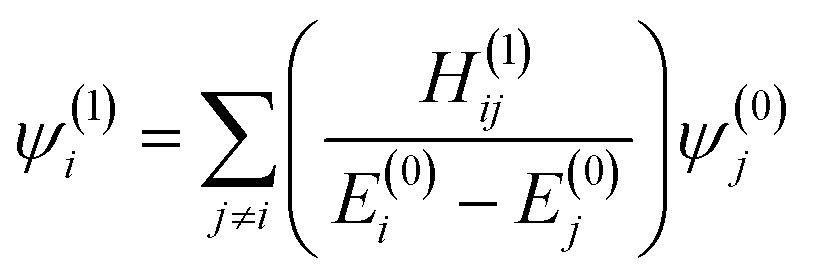
where

2 *H*(1)*ij* = *ψ*(0)*i*|*H*^(1)^|*ψ*(0)*j*

and *H* ^(1)^ is the first order correction to the Hamiltonian. *ψ*(1)*i* is the perturbed wavefunction and can be viewed as a superposition of the unperturbed states of *ψ*(0)*i* where the state *j* will make no contribution if *H*(1)*ij* = 0 and can only make a significant contribution when *E*(0)*i* – *E*(0)*j* is small.

The form of an electric field represented as a multipole expansion from the extended protein environment goes as

3 *H*^(1)^ = *M* + *μ̂* + *Q* +…

where *M* is a monopole term, *μ̂* is the dipole operator, and *Q* is the quadrupole operator, which are all due to the addition of the amino acid residues beyond the base model.

The result of eqn (2) for each term in eqn (3) must contain the totally symmetric irreducible representation of the group in order to be non-zero. For a high symmetry system, the monopole operator is fully symmetric and thus most strongly couples MOs that are of the same symmetry and hence have the same nodal character. In a system lacking symmetry, such as our system, while all *ψ*(0)*j* may couple, those of similar nodal character will couple most strongly. The dipole operator on the other hand is antisymmetric with respect to a nodal plane and most strongly couples orbitals that have different nodal character. For the example of a multipole expansion centered around Zn^2+^, the dipole operator will preferentially couple one-electron orbitals that are of the same phase on one side of Zn^2+^ and of different phase on the other side of the ion. The resulting perturbed wavefunctions will have different nodal character around the zinc atom which will in turn change where ∇*ρ* = 0, moving the positions of critical points.

**Fig. 6:**
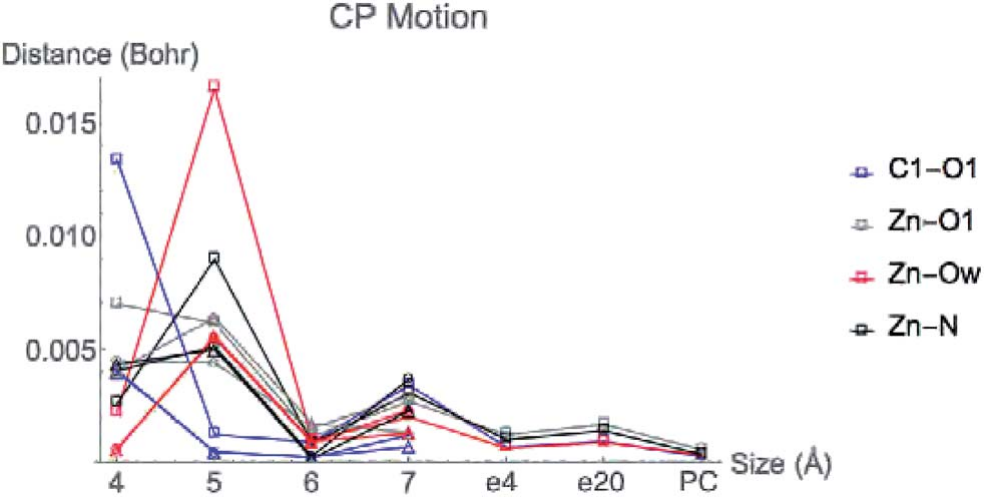
Movement of the CPs as the active site cluster model size increases. Points are plotted for the motion between each size step, *i.e.* points at the 5 Å *x*-value indicate the difference in coordinates of a CP from 4 to 5 Å. All data is from LDA TZP calculations from all configurations with the following shapes: configuration 1-square, 2-circle, 3-triangle. Configuration 1 includes points for changes from 7 Å system with dielectric constants of 4 (e4) and 20 (e20) and with point charges (PC).

**Fig. 7:**
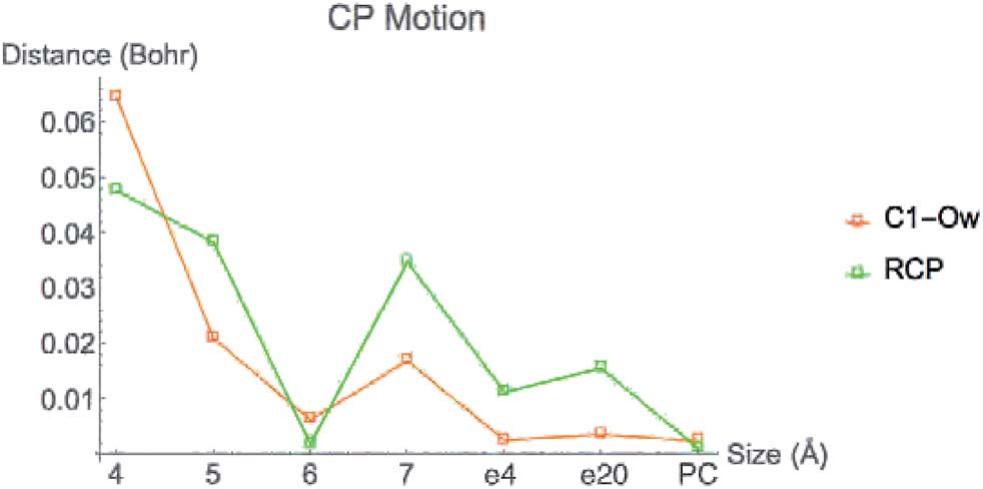
Movement of C1–Ow bond CP and ring CP from the configuration 1 LDA TZP calculation including changes from the 7 Å system with dielectric constants of 4 (e4) and 20 (e20) and with point charges (PC).

Fig. 6 displays the change in position of C1–O1, Zn^2+^–O1, Zn^2+^–Ow, and Zn^2+^–NHis bond CPs for all three nuclear configurations as the system size increases for LDA TZP calculations. The motion of the C1–Ow bond CP and ring CP for configuration 1 is shown in a separate plot in Fig. 7 due to the larger distance scale for these two points. CP distance is measured as the distance between the NÅ and N–1 Å CP position where all atomic coordinates are held fixed for *N* = 4–7. Data from all calculation methods is included in Fig. S5–S7 (ESI†).

In general, the CPs in configuration 1 move greater distances as the system size changes than the points in configurations 2 and 3. For all sizes, the C1–Ow and ring CP have a larger change in their positions than the other CPs tested. As mentioned previously, the charge density in the region of these two critical points is nearly flat so a slight change in nuclear coordinates may annihilate the ring and C1–Ow bond CPs. We have observed that the susceptibility for CP motion is larger in regions of flat charge density since the gradient of *ρ* is small in these areas,^27^ indicating that the system may be approaching a topological catastrophe.^28^ Flat regions of charge density are also more likely to be affected by protein motions, which can play a vital role in the electrostatic environment in the active site.^11,29^

Most of the bond critical points in Fig. 6 have their greatest movement between 3–4 Å or 4–5 Å. However, there is still movement of the critical points as the system size continues to increase. The CPs tested here actually move further as the system size changes from 6–7 Å than from 5–6 Å suggesting that the exact positions of the CPs have not converged in the 7 Å system. Increasing the system size from 5–6 Å adds one amino acid residue for configurations 2 and 3 and four residues for configuration 1. Going from 6–7 Å on the other hand, adds eight residues to all three configurations and a potassium ion to configurations 1 and 2. Also of note is that amino acids are not added symmetrically around the zinc center as the model size increases. The position of each residue, especially charged and polar species, is expected to be related to the variation in CP position. From 6–7 Å, the model without the potassium ion, configuration 3, has less of a change in the CP motion than the other two configurations. The importance of the potassium ion to electrostatic preorganization and CP position is examined in more detail in Section 3.4.

Adding COSMO solvation or point charges to the 7 Å configuration 1 calculation does move the position of critical points, but has less of an effect than changing the cluster model size from 6–7 Å. The small change in the location of critical points when approximating electrostatic effects from the full protein could have multiple causes. One possibility is that the location of CPs is nearly converged for the 7 Å system, meaning there will be little movement when adding in the effect of any more of the protein. A more likely explanation is that point charges and single dielectric constants do not affect the location of point charges to the same degree that using the actual charge density of the full protein would. The influence of *ρ* in the central region by the full charge density of the protein rather than point charges has been emphasized in a QM/MM study of partial charge convergence for catechol *O*-methyltransferase.^30^

While the positions of CPs in the central regions have not converged for the cluster model sizes tested here, none of the distances the CPs move between models are larger than 0.075 bohr, and most are less than 0.010 bohr. However, even slight changes in CP position have been related to significant changes in properties. Eberhart showed that small changes to atomic basin surfaces (which are influenced by CP positions) can have a significant effect on atomic energies.^31^ Jones *et al.* used the distance between critical points as an order parameter for an isostructural phase transition in calcium.^27^ The slight changes in positions of CPs that occur in HDAC8 due to the extended protein environment have a significant effect on activation energies and enzymatic rates, as we showed previously.^16^

### 3.4 Effect of K^+^

The orientation and magnitude of an electric field in a protein will be most affected by polar and charged molecules and ions. In HDAC8, there are two cations near the active site. The first is the Zn^2+^ that is in the central region. The second is a K^+^ ion, which is included in the 7 Å models for configurations 1 and 2, but is not included in any configuration 3 models. The CPs in the central region move less when the model size changes from 6–7 Å for configuration 3 compared to the other two models. The effect of K^+^ on the electrostatics in the active site is undeniable, and in a way represents the most graphical model for what the environment can do to the features of the charge density. We therefore use this K^+^ to particularly illustrate the point of this work. We want to know which features of the charge density are affected by K^+^. It is enchanting that Nature placed such an electrostatic bomb right next to the active site. What exactly is the reason?

**Fig. 8:**
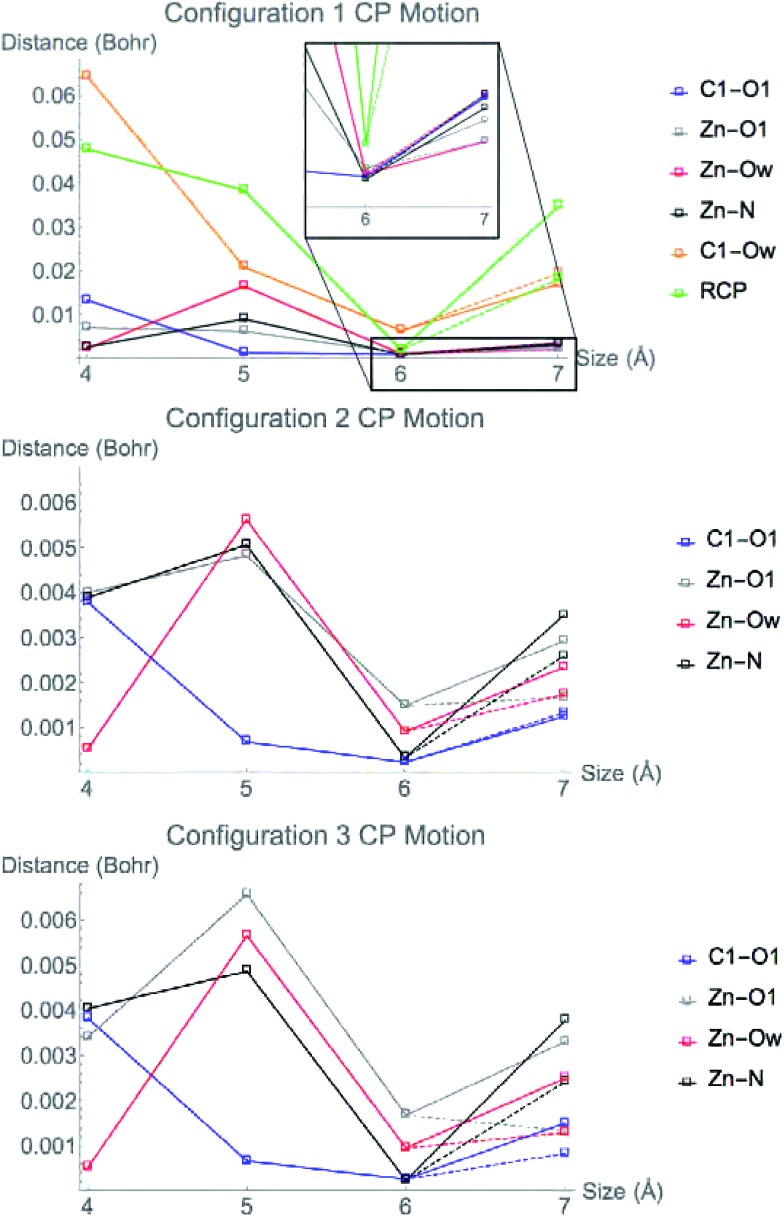
Movement of CPs from the configuration 1 (top), 2 (middle), and 3 (bottom) LDA TZP calculation with (solid lines) and without (dashed lines) K^+^ included in the 7 Å cluster model.

We investigated the effect the potassium ion has on CP motion in the central region by calculating the charge density for all three of the 7 Å systems, both with and without the K^+^ ion included in the simulation. Fig. 8 shows the change in CP motion for the three systems. For configuration 3, all CPs in the central region move a greater distance when K^+^ is included in the simulation, indicating that the ion does indeed have a large influence on the electrostatic environment in the active site. For configuration 2, all of the CPs except for the C1–O1 bond CP move a greater distance when the K^+^ is included. However, in configuration 1, only the ring CP and Zn^2+^–O1 bond CP move a larger distance with K^+^. The C1–Ow, Zn^2+^–N, Zn^2+^–Ow CP move less when K^+^ is included and the C1–O1 has no difference in its motion with or without K^+^.

The Zn^2+^–O1 bond CP has greater motion when K^+^ is included in the model for all nuclear configurations tested. This bonding interaction is critical for the substrate to bind to the active site of HDAC8. When performing QM/DMD simulations for ref. 16, the substrate molecule lost coordination to the Zn^2+^ if the potassium ion was not included in the QM region. These observations suggest that the potassium ion is vital to the proper electrostatic environment in the active site for substrate binding to occur.

## 4 Discussion

The magnitude of *ρ* in the central region of HDAC8 is directly influenced by the local environment, which we find here to be represented by a cluster model that includes a full residue sphere around the point of interest. Rapid convergence of the magnitude of *ρ* was expected since the charge density is known to be near-sighted and decay exponentially.^32^ This has also been observed for organic molecules with varying functional groups.^33^

The sensitivity of the position of active site CPs due to the addition of atoms far away indicates that CP position is due to perturbations to the wavefunction in the active site caused by the protein's extended environment.

Studies have shown similar magnitudes of changes in position of bond CPs on small organic molecules due to the addition of electron withdrawing and donating groups.^34,35^ Perturbations to wavefunctions that move CPs are due primarily to the orientations and positions of dipole moments from each atom in the surrounding protein, and will be most prominent for charged and polar residues, including the K^+^ present in the 7 Å model for configurations 1 and 2.

The distances between CPs, as well as the curvature of the charge density at these points, can also be related to reaction coordinates through the electron preceding picture presented by Ayers *et al.* ^36–38^ In the rate-determining step of the deacetylation reaction, ts2, the ring CP must move towards one of the Zn^2+^–O bond CPs to annihilate both CPs, opening the tetrahedral ring structure pictured in Fig. 1. The reaction barrier for this step will be lower when the path through the charge density between the two points is least steep, since it costs less energy to change the charge density by small amounts than large amounts, as has been demonstrated in the case of a computationally designed carboxypeptidase A.^39^ The directions and magnitudes of curvature of *ρ* at CPs involved in the reaction step can be used to determine how a reaction will proceed.^38^ A flat charge density between CPs can occur through two methods, either by a change in the magnitude of *ρ* at the CPs, or by a change in the distance between the two points. Here we find the magnitude of *ρ* at CPs to be directly affected by the local environment and not the full protein. The positions of CPs, however, are influenced by the extended environment of the protein. Electrostatic preorganization may be causing the CPs in the active site to move into the most favorable positions in order to create a flat charge density along the reaction coordinate. We propose that the exact positions of CPs can be used as a measure of electrostatic preorganization, which is also influenced by polar and charged species.

To test this theory, a non-metal enzyme that catalyzes a simple one-step reaction would be preferred. In this case, the direction of the motion of CPs necessary for the reaction to occur would be clear. For HDAC8, the rate determining step is the second step, so it is difficult to determine the preferred direction for the CPs to move in the enzyme–reactant complex to facilitate catalysis and increase the reaction rate. Furthermore, the fact that HDAC8 has two metal cations near the active site may make the effects of electrostatic preorganization from the rest of the protein less important, as K^+^ has been shown to have a large influence on the electrostatic environment in the central region. In an electrostatically preorganized active site, it should not only be easy for the critical points directly participating in a reaction to move, but the CPs surrounding the active site should show the opposite behavior and be difficult to move. When an enzyme is electrostatically preorganized, the dipoles around the active site should not change throughout the reaction coordinate as has been demonstrated by Boxer *et al.* ^8^ CPs surrounding the active site with high curvatures (non-flat regions of *ρ*) should be more difficult to move and could be used to predict the preorganization of an active site, especially for enzyme design.

## 5 Conclusions

Electrostatic preorganization is considered to be the dominant factor in the high catalytic efficiencies of enzymes. Quantifying the effect of the protein's extended environment on the active site electrostatics is a challenging problem, but is vital to improving enzyme design and the general understanding of enzyme structure and kinetics. We have tested the convergence of the geometry of the electron charge density in the active site of Histone Deacetylase 8 as increasing amounts of the surrounding protein are included in a cluster model. The magnitude of the charge density at critical points is found to be dependent on the local environment and converges once one residue sphere is added around the point of interest. The Bader atomic charges of atoms usually converge at this same limit, with slight variations in charge values for some atoms at larger size models. Adding a dielectric constant or point charges to the simulation has a slight effect on atomic charges, but does not alter the converged values for the magnitude of *ρ* at CPs.

The positions of critical points, however, are generally not converged up to the limit of active site model sizes tested here and are shown to be affected by the extended environment of the protein. This finding elucidates the effect of electrostatic preorganization on the charge density. It is not the charges on atoms, or the nature of the bonding interactions, but the relative positions of CPs that reveals the signature of electrostatic preorganization. The locations of critical points are influenced by dipole moments from residues in the full protein, which manifest as perturbations to the wavefunction in the active site. Critical point positions are known to correlate with reactivity and reaction barriers. The positions and curvatures of the charge density at CPs could then be used to predict the extent of electrostatic preorganization in computationally designed enzymes.
