# Supplementary material for: Quantified electrostatic preorganization in enzymes using the geometry of the electron charge density
Source: Chem Sci. 2017 Apr 24;8(7):5010–8. doi: 10.1039/c7sc01301a (PMC5612031; doi:10.1039/c7sc01301a)
Supplement: Supplementary file 1 [file SC-008-C7SC01301A-s001.pdf]

Electronic Supplementary Material (ESI) for Chemical Science.
This journal is © The Royal Society of Chemistry 2017

Quantified Electrostatic Preorganization in Enzymes Using the Geometry of the Electron Charge Density
Electronic Supplementary Information

Amanda Morgenstern,† Matthew Jaszai,† Mark E. Eberhart,*,† and Anastassia N. Alexandrova*,‡

†*Molecular Theory Group, Colorado School of Mines*

‡*Department of Chemistry and Biochemistry, University of California, Los Angeles*

E-mail: meberhar@mines.edu; ana@chem.ucla.edu

Quantum Theory of Atoms in Molecules

The use of critical point (CP) analysis as a means to describe the structure of an evolving charge density, ρ, was first articulated by Bader as part of his Quantum Theory of Atoms in Molecules (QTAIM).[1,2] Central to this theory is a representation of the charge density in terms of the elements of its critical set. The CPs are the zero-dimensional elements of this set and coincide with points where the gradient of the charge density, $\nabla\rho$, vanishes. There are four types of CPs classified by the eigenvalues of the Hessian of ρ at the CP. CPs of saddle character with two negative curvatures (bond CPs) and one-dimensional lines (bond paths) are associated with bonding interactions. Ring CPs are saddle points with one negative curvature and occur inside rings of bond paths, such as inside a benzene ring. Cage

CPs, minima in ρ, exist inside cages of atoms connected by bond paths, such as in fullerene. Two dimensional surfaces of zero-flux in the gradient of the charge density, $\nabla\rho$, surrounding nuclear CPs (maxima) delineate the boundaries of individual atoms in molecules. Atoms in molecules, or atomic basins, are three-dimensional structures with well-defined energies and properties that depend on the energy.

Among the properties associated with an atom is its Bader charge, defined as the difference between the integrated electron density over an atomic basin and the atom's atomic number. There are many methods for calculating atomic charges with some common choices being Mulliken,[3,4] Hirshfeld,[5] and natural population analysis (NPA).[6] We choose to only report Bader charges in this work due to the focus on the electron charge density. Methods such as Mulliken charges and NPA rely on wavefunctions and are often highly basis set dependent. Hirshfeld charges are based on the charge density, but require a promolecular charge density for comparison. Bader charges are based only on the topology and geometry of the charge density of the molecule in question. For a useful review, see Ref. 7.

Residues in Each Size Model

Table S1: Amino acid residues and metal ions included in active site models from the three QM/DMD configurations and total number of residues and atoms for each configuration for the 7Å model.

	Config. 1	Config. 2	Config. 3
3Å system	Asp178	Asp178	Asp178
	His180	His180	His180
	Asp267	Asp267	Asp267
	Zn^{2+}	Zn^{2+}	Zn^{2+}
	Substrate	Substrate	Substrate
4Å system	Leu179	Leu179	Leu179
	Gly304	Tyr306	Tyr306
	Tyr306		
5Å system	His 142	His142	His142
	His143	His143	His143
	Gln263	Gly304	Gly304
6Å system	Gly265	Gly303	Gly303
	Met274		
	Gly303		
	Gly305		
7Å system	Ala266	Cys153	Cys153
	Thr268	His181	His181
	His181	Pro209	Pro209
	Phe207	Gly265	Gly265
	Phe208	Ala266	Ala266
	Gly151	Thr268	Thr268
	Phe152	Met274	Met274
	Asp176	Gly305	Gly305
	K^+	K^+	
Residues	21	17	17
Atoms	352	277	276

Topology of Active Site for Three Nuclear Configurations

The topology of the charge density in the central region of HDAC8 varies depending on the nuclear coordinates chosen from QM/DMD simulations. The bond paths and critical points in the central region for the three lowest energy configurations studied are shown in

Figure S1. The lowest energy coordinates, configuration 1, possess a bond path between the carbonyl carbon (C1) and the water oxygen (Ow), which also necessitates the existence of a ring CP. This bond path is not present in configurations 2 or 3.

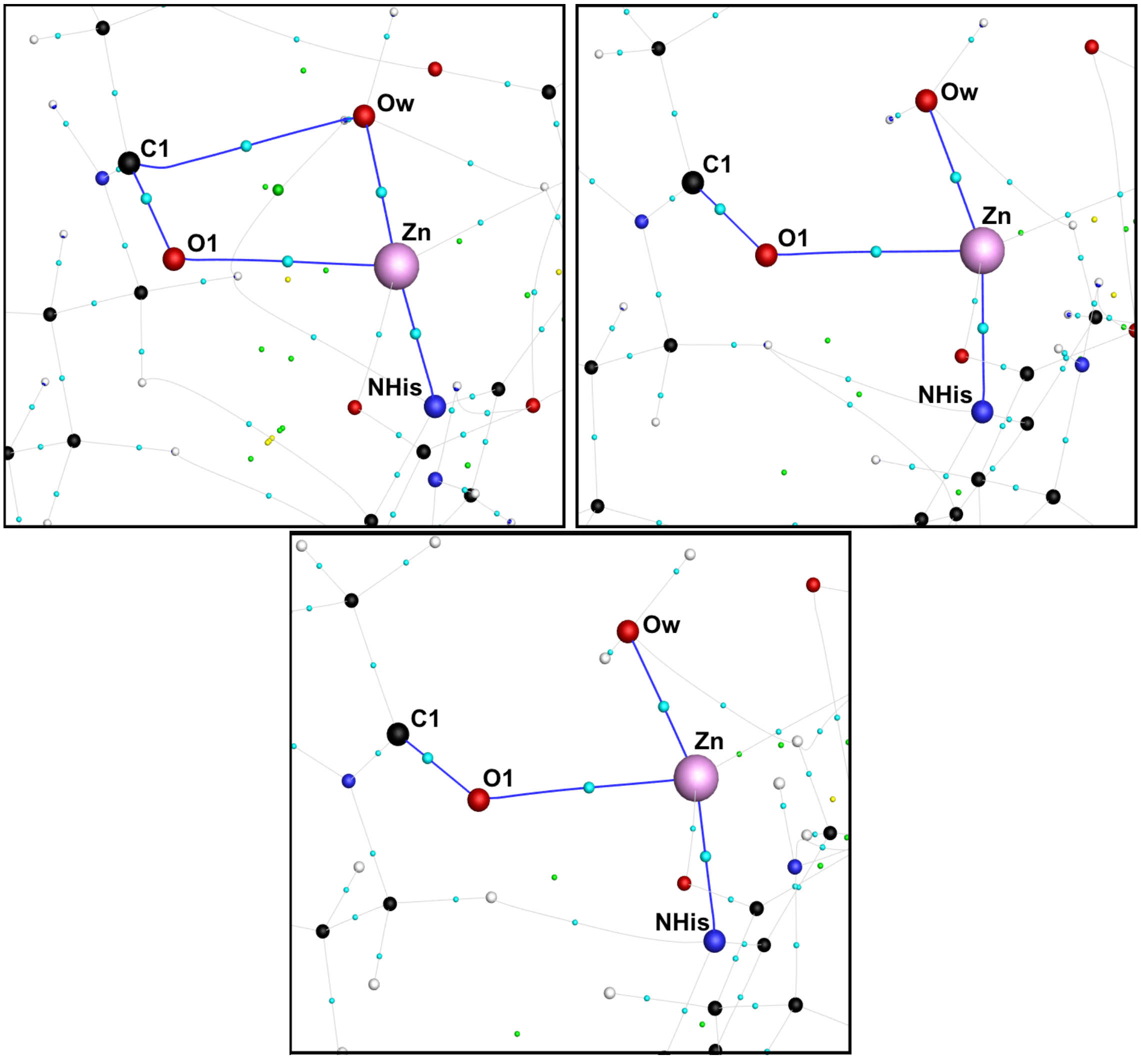

Figure S1: Topology in the central region of HDAC8 for configuration 1 (top left), 2 (top right), and 3 (bottom). Bond CPs are shown in cyan and ring CPs in green.

Bader Atomic Charges

In general, atomic charges do not vary much as the basis set switches from DZP to TZP, but the functional choice can make a difference in the charges. Even the relative differences

between charges for different atoms are dependent on functional choice in some cases. For example, Using an LDA functional, the Ow charge is lower than the O1 charge for system sizes between 5–7Å but for the M06L functional the configuration 1 and configuration 2 Ow charge is similar to O1. However, most of the trends of atomic charges are consistent as system size increases.

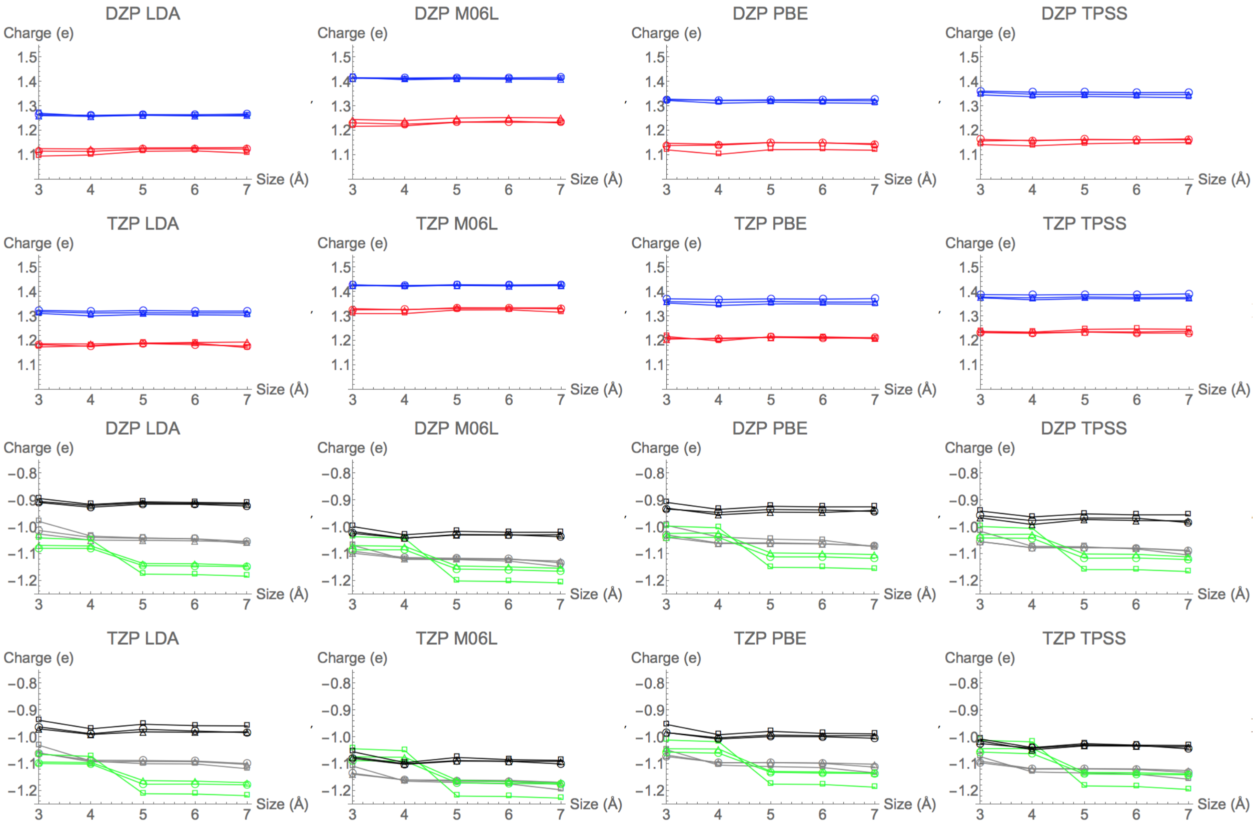

Figure S2: Bader atomic charges for Zn^{2+} (blue), C1 (red), O1 (grey), Ow (green), and NHis (black). Each plot shows results for one calculation method as active site model sizes increase from 3–7Å. The three lines for each atom on each plot represent the three different starting QM/DMD configurations used with the following shapes: configuration 1-square, 2-circle, 3-triangle.

Bader Atomic Volumes

The volumes of Bader atomic basins using a cut-off of $\rho = 0.001$ for nuclear configuration 1 are displayed in Figure S3. Volumes for Ow and O1 initially decrease as the system size

modeled increases to 4 and 5Å. This is due to the full residue sphere being added to the truncated protein model around these atoms, which drastically reduces their volumes. Once the 5Å system size is reached, the volumes of the five atomic basins mostly change inversely to the atomic charges presented in the main text (bottom of Figure S3). This is expected, as a lower charge means more electrons are added to the atomic basin, resulting in a larger volume. As with atomic charges, the volumes of atoms are mostly converged at the 5Å model size, except for O1 and Ow.

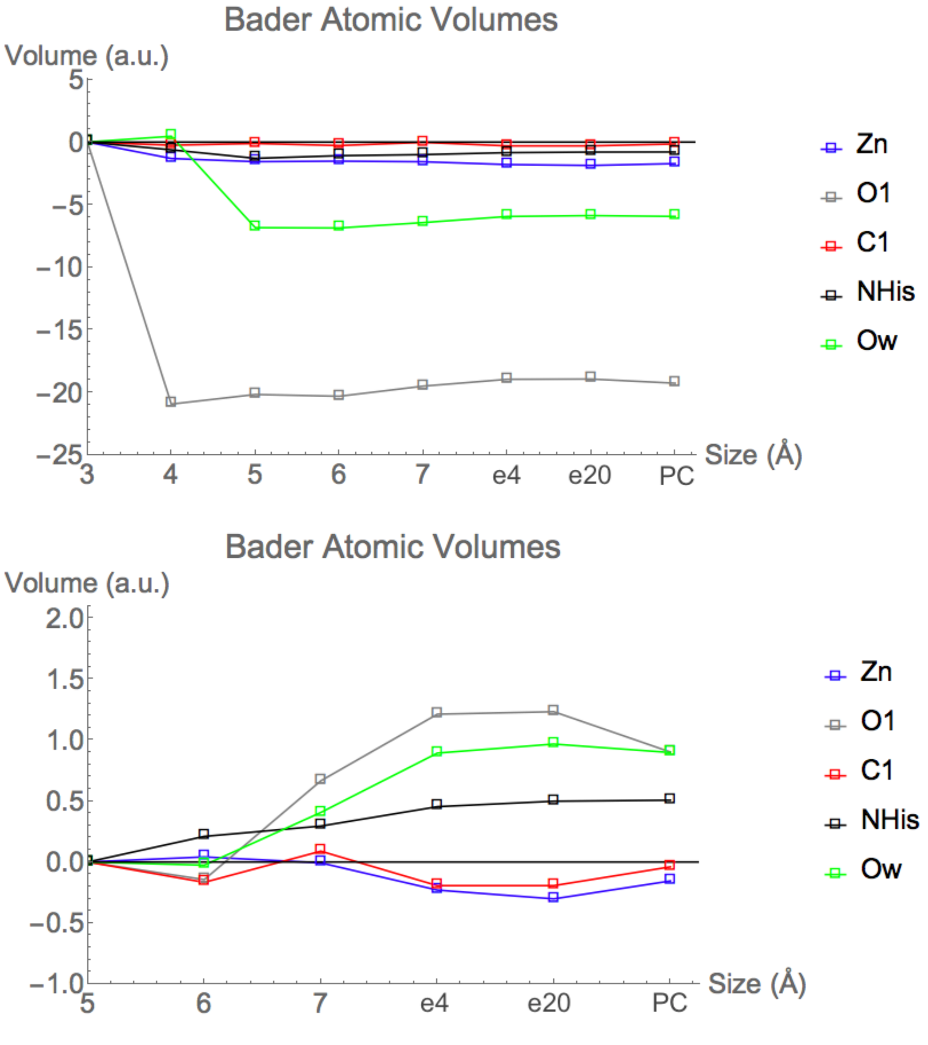

Figure S3: Bader atomic volumes for Zn^{2+}, O1, C1, NHis, and Ow using the coordinates from nuclear configuration 1. The bottom figure shows the deviation in volume for each atom from the 5Å system atom volumes—once a full residue sphere is included around the five atoms tested.

Magnitude of ρ at CPs

There are small variations in the amount of charge density at critical points based on method choice, but the trends as system size increases are consistent across all functionals and basis sets.

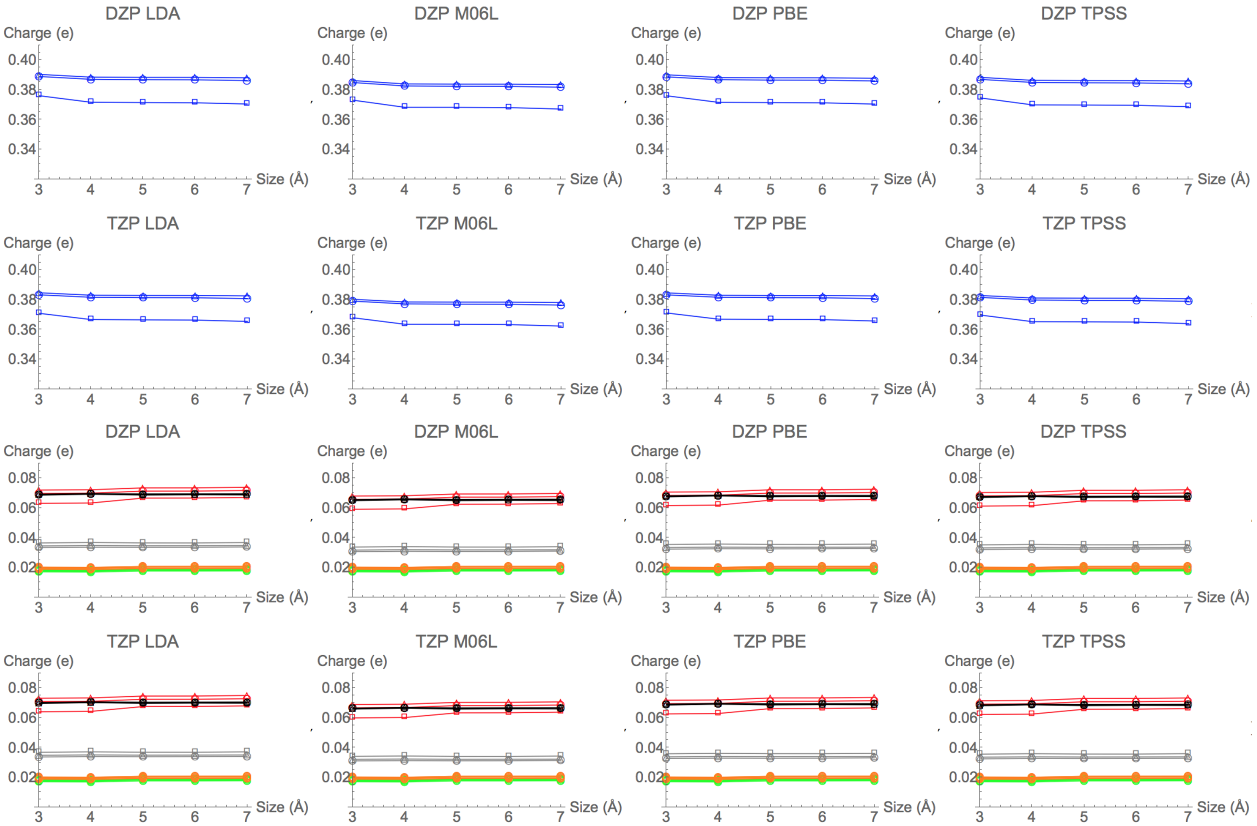

Figure S4: Charge density at critical points for C1–O1 (blue), C1–Ow (orange), Ring CP (green), Zn^{2+}–O1 (grey), Zn^{2+}–Ow (red), and Zn^{2+}–N (black). Each plot shows results for one calculation method as active site model sizes increase in size from 3–7Å. The three lines for each atom on each plot represent the three different starting QM/DMD configurations used with the following shapes: configuration 1-square, 2-circle, 3-triangle.

Critical Point Motion

The motion of CPs is less dependent on functional or basis set choice than atomic charges and the values of ρ at CPs. The method of calculation has a smaller influence on the exact position of each CP than the nuclear configuration used or the size of model. The only

instance where the basis set has an impact on the trends of positions of CPs as system size increases is for 3–4Å and 4–5Å for the Zn–O1 bond CP movement using nuclear configuration 2 (Figure S6). The functional choice slightly changes the magnitude of distances moved in some cases, but does not change any trends in CP motion as system size changes.

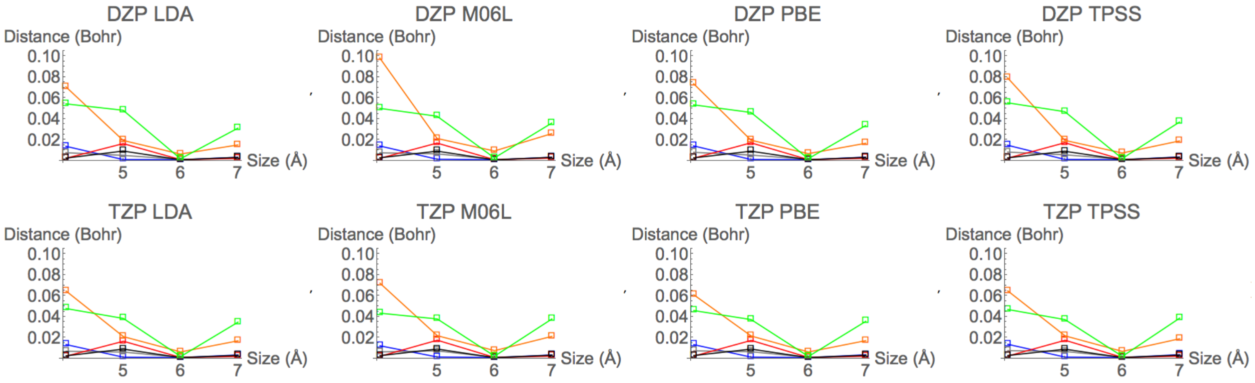

Figure S5: Movement of the CPs as the active site cluster model size increases for nuclear configuration 1 with all calculation methods using the following coloring: C1–O1 (blue), Zn^{2+}–O1 (grey), Zn^{2+}–Ow (red), Zn^{2+}–N (black), C1–Ow (orange), and Ring CP (green).

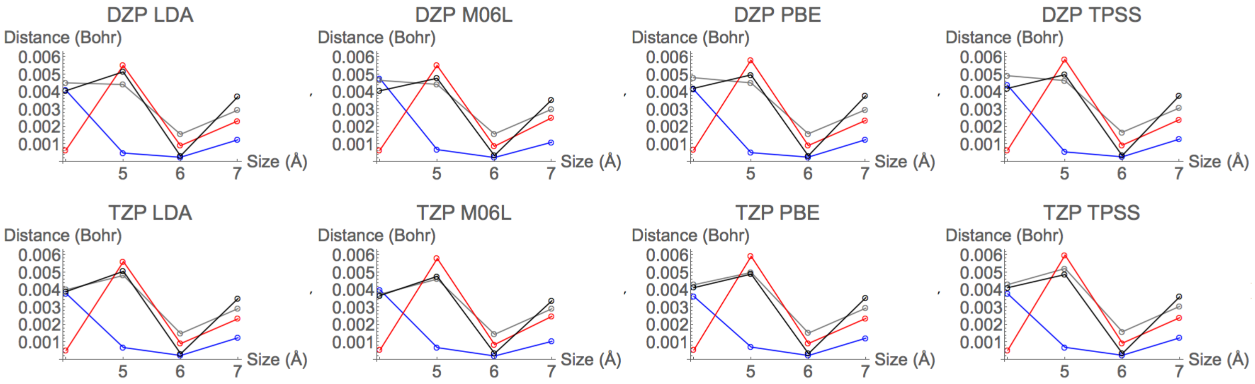

Figure S6: Movement of the CPs as the active site cluster model size increases for nuclear configuration 2 with all calculation methods using the following coloring: C1–O1 (blue), Zn^{2+}–O1 (grey), Zn^{2+}–Ow (red), and Zn^{2+}–N (black).

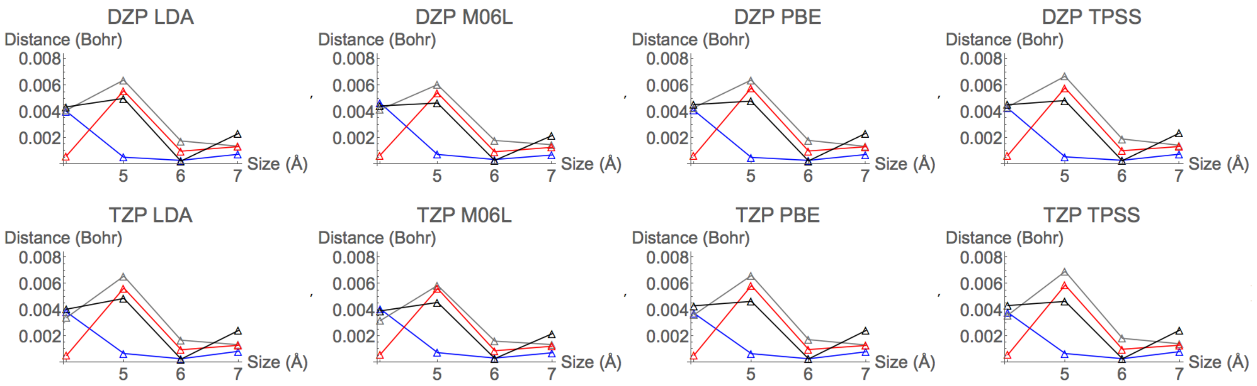

Figure S7: Movement of the CPs as the active site cluster model size increases for nuclear configuration 3 with all calculation methods using the following coloring: C1–O1 (blue), Zn^{2+}–O1 (grey), Zn^{2+}–Ow (red), and Zn^{2+}–N (black).

References

(1) Bader, R. F. W. *Acc. Chem. Res.* **1985**, *18*, 9–15.

(2) Bader, R. F. W. *Atoms in Molecules: A Quantum Theory*; Clarendon Press: Oxford, UK, 1990.

(3) Mulliken, R. S. *J. Chem. Phys.* **1955**, *23*, 1833.

(4) Mulliken, R. S. *J. Chem. Phys.* **1955**, *23*, 1841.

(5) Hirshfeld, F. L. *Theor. Chem. Acc.* **1977**, *44*, 129–138.

(6) Reed, A. E.; Weinstock, R. B.; Weinhold, F. *Journal of Chemical Physics* **1985**, *83*, 735–746.

(7) Guerra, C. F.; Handgraaf, J.-W.; Baerends, E. J.; Bickelhaupt, F. M. *J. Comput. Chem.* **2004**, *25*, 189–210.

Nuclear Coordinates for 7 Angstrom HDAC8 model sizes for all three nuclear configurations obtained from QM/DMD simulations

Configuration 1

1 N	96.548000000000	96.155000000000	102.798000000000
2 C	95.428000000000	96.182000000000	101.840000000000
3 C	94.675000000000	94.901000000000	101.553000000000
4 O	93.625000000000	94.913000000000	100.894000000000
5 C	95.941000000000	96.747000000000	100.516000000000
6 C	97.087000000000	95.982000000000	99.941000000000
7 N	97.455000000000	96.122000000000	98.614000000000
8 C	98.005000000000	95.097000000000	100.487000000000
9 C	98.545000000000	95.350000000000	98.402000000000
10 N	98.912000000000	94.724000000000	99.518000000000
11 H	97.445130110000	96.502133460000	102.451165800000
12 H	97.062157560000	96.854102910000	97.870033780000
13 N	95.181000000000	93.774000000000	102.026000000000
14 C	94.467000000000	92.499000000000	101.868000000000
15 C	93.350000000000	92.102000000000	102.829000000000
16 O	92.581000000000	91.199000000000	102.542000000000
17 C	95.422000000000	91.315000000000	101.852000000000
18 C	96.536000000000	91.483000000000	100.866000000000
19 N	96.297000000000	91.927000000000	99.585000000000
20 C	97.894000000000	91.190000000000	100.902000000000
21 C	97.467000000000	91.895000000000	98.905000000000
22 N	98.470000000000	91.445000000000	99.667000000000
23 H	96.058170860000	93.845790420000	102.568616300000
24 H	95.423517480000	92.307733440000	99.201333880000
25 N	97.168000000000	87.802000000000	103.867000000000
26 C	98.325000000000	88.403000000000	103.180000000000
27 C	99.152000000000	89.304000000000	104.047000000000
28 O	99.670000000000	90.369000000000	103.683000000000
29 H	96.901578970000	86.858184440000	103.581152600000
30 N	99.356000000000	88.849000000000	105.304000000000
31 C	100.134000000000	89.615000000000	106.284000000000
32 C	99.387000000000	90.802000000000	106.934000000000
33 O	99.936000000000	91.519000000000	107.745000000000
34 C	101.535000000000	90.076000000000	105.899000000000
35 C	102.264000000000	89.059000000000	105.063000000000
36 C	101.981000000000	87.701000000000	105.148000000000
37 C	103.214000000000	89.496000000000	104.138000000000
38 C	102.722000000000	86.804000000000	104.373000000000
39 C	103.909000000000	88.596000000000	103.326000000000
40 C	103.680000000000	87.228000000000	103.445000000000
41 H	98.779328760000	88.040132730000	105.586090100000

42 N	97.055000000000	101.761000000000	94.153000000000
43 C	97.751000000000	100.702000000000	94.846000000000
44 C	98.886000000000	100.180000000000	93.979000000000
45 O	98.611000000000	99.512000000000	92.984000000000
46 C	96.796000000000	99.551000000000	95.154000000000
47 C	97.406000000000	98.478000000000	96.057000000000
48 O	98.630000000000	98.172000000000	95.882000000000
49 O	96.602000000000	97.926000000000	96.865000000000
50 H	96.662341190000	101.523313800000	93.235980000000
51 N	101.532000000000	98.418000000000	95.148000000000
52 C	102.213000000000	97.244000000000	95.708000000000
53 C	102.208000000000	96.107000000000	94.640000000000
54 O	101.364000000000	96.099000000000	93.731000000000
55 C	101.518000000000	96.920000000000	97.058000000000
56 C	101.963000000000	95.655000000000	97.783000000000
57 O	102.421000000000	95.686000000000	98.948000000000
58 O	101.794000000000	94.543000000000	97.150000000000
59 H	100.577133400000	98.576634930000	95.502462710000
60 N	103.160000000000	95.154000000000	94.721000000000
61 C	103.171000000000	94.058000000000	93.756000000000
62 C	101.890000000000	93.198000000000	93.700000000000
63 O	101.595000000000	92.579000000000	92.668000000000
64 C	104.318000000000	93.140000000000	94.174000000000
65 C	104.675000000000	91.891000000000	93.369000000000
66 C	105.115000000000	92.264000000000	91.956000000000
67 C	105.713000000000	91.046000000000	94.089000000000
68 H	103.821859100000	95.097940650000	95.532326270000
69 N	101.201000000000	93.094000000000	94.866000000000
70 C	100.032000000000	92.251000000000	94.991000000000
71 C	98.743000000000	93.069000000000	94.751000000000
72 O	98.702000000000	94.294000000000	94.927000000000
73 C	99.990000000000	91.583000000000	96.394000000000
74 C	101.209000000000	90.764000000000	96.700000000000
75 N	102.338000000000	91.303000000000	97.309000000000
76 C	101.454000000000	89.428000000000	96.441000000000
77 C	103.237000000000	90.327000000000	97.411000000000
78 N	102.735000000000	89.176000000000	96.899000000000
79 H	101.430594800000	93.739659130000	95.661547500000
80 H	103.280262300000	88.315126130000	96.769103150000
81 N	97.648000000000	92.390000000000	94.326000000000
82 C	96.411000000000	93.106000000000	94.031000000000
83 C	95.810000000000	93.761000000000	95.279000000000
84 O	95.828000000000	93.216000000000	96.383000000000
85 C	95.450000000000	92.096000000000	93.341000000000

86 C	94.028000000000	92.551000000000	93.188000000000
87 N	92.963000000000	91.818000000000	93.689000000000
88 C	93.488000000000	93.643000000000	92.606000000000
89 C	91.843000000000	92.456000000000	93.417000000000
90 N	92.139000000000	93.567000000000	92.758000000000
91 H	97.715687770000	91.386376240000	94.117838990000
92 H	93.060699490000	90.927605960000	94.187748960000
93 N	99.661000000000	83.078000000000	91.919000000000
94 C	100.317000000000	84.222000000000	92.563000000000
95 C	99.289000000000	84.979000000000	93.427000000000
96 O	98.245000000000	85.434000000000	92.908000000000
97 C	100.876000000000	85.211000000000	91.541000000000
98 C	101.807000000000	86.255000000000	92.166000000000
99 C	103.043000000000	85.925000000000	92.715000000000
100 C	101.291000000000	87.524000000000	92.413000000000
101 C	103.824000000000	86.890000000000	93.329000000000
102 C	102.060000000000	88.504000000000	93.060000000000
103 C	103.339000000000	88.177000000000	93.533000000000
104 H	98.841702540000	83.328084790000	91.352667970000
105 N	99.673000000000	85.226000000000	94.653000000000
106 C	98.743000000000	85.837000000000	95.627000000000
107 C	98.273000000000	87.219000000000	95.215000000000
108 O	99.088000000000	87.997000000000	94.745000000000
109 C	99.438000000000	85.928000000000	97.012000000000
110 C	98.535000000000	86.307000000000	98.163000000000
111 C	97.806000000000	85.327000000000	98.853000000000
112 C	98.376000000000	87.628000000000	98.538000000000
113 C	96.991000000000	85.699000000000	99.921000000000
114 C	97.525000000000	88.010000000000	99.576000000000
115 C	96.814000000000	87.042000000000	100.259000000000
116 H	100.608573200000	84.956553560000	94.978143110000
117 N	98.993000000000	103.680000000000	98.071000000000
118 C	99.637000000000	102.560000000000	98.709000000000
119 C	100.885000000000	102.176000000000	97.903000000000
120 O	100.873000000000	102.087000000000	96.695000000000
121 C	98.669000000000	101.367000000000	98.807000000000
122 C	99.036000000000	100.333000000000	99.854000000000
123 C	100.027000000000	99.280000000000	99.374000000000
124 O	100.729000000000	99.500000000000	98.386000000000
125 N	100.055000000000	98.117000000000	100.034000000000
126 H	98.995828650000	103.652166700000	97.045865180000
127 H	100.670041600000	97.356539660000	99.707419570000
128 H	99.392341930000	97.898858040000	100.780263100000
129 N	103.564000000000	99.869000000000	98.257000000000

130 C	104.370000000000	98.712000000000	98.727000000000
131 C	105.652000000000	98.569000000000	97.910000000000
132 O	105.624000000000	98.633000000000	96.680000000000
133 H	102.543915100000	99.716543480000	98.244710510000
134 N	106.766000000000	98.349000000000	98.569000000000
135 C	108.044000000000	98.224000000000	97.833000000000
136 C	108.508000000000	96.765000000000	97.552000000000
137 O	109.656000000000	96.527000000000	97.122000000000
138 C	109.183000000000	98.825000000000	98.846000000000
139 H	106.755434300000	98.097860570000	99.579311830000
140 N	107.523000000000	95.856000000000	97.473000000000
141 C	107.800000000000	94.504000000000	97.003000000000
142 C	107.852000000000	94.409000000000	95.499000000000
143 O	108.133000000000	93.359000000000	94.937000000000
144 C	106.842000000000	93.436000000000	97.571000000000
145 C	105.360000000000	93.835000000000	97.489000000000
146 O	105.016000000000	94.753000000000	96.713000000000
147 O	104.576000000000	93.130000000000	98.240000000000
148 H	106.535562700000	96.109234030000	97.626116110000
149 N	107.825000000000	95.563000000000	94.821000000000
150 C	108.026000000000	95.633000000000	93.362000000000
151 C	109.491000000000	95.871000000000	93.018000000000
152 O	109.891000000000	95.834000000000	91.837000000000
153 C	107.275000000000	96.841000000000	92.780000000000
154 O	107.657000000000	98.022000000000	93.472000000000
155 C	105.771000000000	96.759000000000	92.957000000000
156 H	107.655834600000	96.462627160000	95.288192050000
157 H	106.884504100000	98.447261510000	93.938649080000
158 N	110.901000000000	88.251000000000	96.579000000000
159 C	109.805000000000	87.802000000000	95.694000000000
160 C	110.129000000000	87.775000000000	94.205000000000
161 O	109.586000000000	86.987000000000	93.460000000000
162 C	108.521000000000	88.571000000000	96.054000000000
163 C	107.861000000000	88.074000000000	97.336000000000
164 S	106.302000000000	88.852000000000	97.632000000000
165 C	105.743000000000	88.007000000000	99.222000000000
166 H	111.085665000000	89.256057190000	96.624844420000
167 N	103.387000000000	99.347000000000	102.679000000000
168 C	102.783000000000	98.019000000000	102.396000000000
169 C	103.778000000000	96.909000000000	102.457000000000
170 O	104.443000000000	96.721000000000	103.488000000000
171 H	103.923879600000	99.350844150000	103.557443800000
172 N	103.916000000000	96.175000000000	101.330000000000
173 C	104.892000000000	95.087000000000	101.245000000000

174 C	106.305000000000	95.627000000000	101.031000000000
175 O	106.543000000000	96.823000000000	100.922000000000
176 H	103.330169700000	96.310881550000	100.484420500000
177 N	107.303000000000	94.724000000000	101.065000000000
178 C	108.707000000000	95.084000000000	100.943000000000
179 C	109.500000000000	94.135000000000	101.846000000000
180 O	109.506000000000	94.272000000000	103.079000000000
181 H	107.045984900000	93.773204090000	101.320454800000
182 N	110.141000000000	93.112000000000	101.242000000000
183 C	110.742000000000	92.032000000000	102.017000000000
184 C	112.234000000000	91.992000000000	101.840000000000
185 O	112.952000000000	91.116000000000	102.350000000000
186 C	110.144000000000	90.689000000000	101.596000000000
187 C	108.660000000000	90.644000000000	101.344000000000
188 C	107.863000000000	91.790000000000	101.163000000000
189 C	108.042000000000	89.383000000000	101.198000000000
190 C	106.510000000000	91.688000000000	100.807000000000
191 C	106.701000000000	89.263000000000	100.832000000000
192 C	105.929000000000	90.420000000000	100.608000000000
193 O	104.654000000000	90.262000000000	100.182000000000
194 H	110.114175200000	93.021767080000	100.219579900000
195 H	104.075158400000	91.139646430000	100.230195400000
196 Zn	102.567000000000	93.107000000000	98.367000000000
197 K	98.847000000000	96.587000000000	93.747000000000
198 H	100.100252100000	92.123546670000	99.355685400000
199 O	103.114000000000	92.332000000000	100.468000000000
200 H	102.498661000000	83.742180530000	102.176603200000
201 C	102.135000000000	92.260000000000	101.270000000000
202 C	101.781000000000	93.458000000000	102.115000000000
203 N	101.413000000000	91.138000000000	101.413000000000
204 H	100.725438800000	91.065377660000	102.188973600000
205 H	100.040401400000	93.768975300000	99.414976960000
206 O	100.717000000000	92.928000000000	99.239000000000
207 C	101.502000000000	89.978000000000	100.517000000000
208 C	101.141000000000	88.693000000000	101.251000000000
209 C	101.471000000000	87.497000000000	100.436000000000
210 C	100.870000000000	86.215000000000	100.977000000000
211 C	101.395000000000	85.077000000000	100.110000000000
212 N	100.845000000000	83.768000000000	100.360000000000
213 H	100.646056800000	83.643658680000	101.368371700000
214 C	102.859000000000	84.821000000000	100.412000000000
215 O	103.680000000000	85.172000000000	99.557000000000
216 H	102.650493100000	93.760610610000	102.726939400000
217 H	101.566219500000	94.289163730000	101.416495300000

218	H	100.906972800000	93.276525080000	102.762116000000
219	H	102.536711200000	89.890926060000	100.141149600000
220	H	100.824974700000	90.129152320000	99.647803980000
221	C	100.186000000000	82.907000000000	99.625000000000
222	O	100.323000000000	82.925000000000	98.419000000000
223	H	100.050821900000	88.709703630000	101.449583200000
224	H	101.665869900000	88.665841190000	102.227626600000
225	H	101.097971700000	87.639500570000	99.396750370000
226	H	102.574891700000	87.392414780000	100.355415500000
227	H	99.759900070000	86.257090640000	100.900659700000
228	H	101.164366500000	86.074519760000	102.040474000000
229	H	101.326743700000	85.303638090000	99.033669710000
230	N	103.177000000000	84.146000000000	101.532000000000
231	H	96.332881480000	96.398458370000	103.769040800000
232	H	94.631637070000	96.876218950000	102.185411000000
233	H	96.221753870000	97.809963860000	100.667767500000
234	H	95.103161060000	96.739319940000	99.790879750000
235	H	98.048797240000	94.685623780000	101.492312100000
236	H	99.036932470000	95.257558600000	97.431961190000
237	H	93.944115700000	92.579356250000	100.888408900000
238	H	93.274399410000	92.668345160000	103.788336400000
239	H	94.819485760000	90.412708310000	101.622950600000
240	H	95.867693680000	91.174458630000	102.858769600000
241	H	98.486825930000	90.896740100000	101.771003000000
242	H	97.531557390000	92.245505530000	97.874381880000
243	H	96.389727980000	88.426886800000	104.092459700000
244	H	98.058975230000	88.966201660000	102.263246400000
245	H	98.982581210000	87.574279320000	102.850218300000
246	H	100.256779300000	88.907880520000	107.137210600000
247	H	98.318308650000	90.934529990000	106.629327800000
248	H	102.084057500000	90.289415970000	106.836380800000
249	H	101.465257000000	91.021381160000	105.331900600000
250	H	101.254663500000	87.315289800000	105.868514400000
251	H	103.422015700000	90.569288710000	104.050170500000
252	H	102.548800300000	85.727704710000	104.502998500000
253	H	104.645995400000	88.988401930000	102.614941800000
254	H	104.226087600000	86.500189980000	102.835189800000
255	H	97.391177560000	102.719425900000	94.267007930000
256	H	98.171467350000	101.105874500000	95.785848720000
257	H	99.925999360000	100.515329400000	94.190018190000
258	H	95.887635630000	99.957657330000	95.627627020000
259	H	103.277511700000	97.496424860000	95.917887330000
260	H	100.427660100000	96.851883750000	96.847163170000
261	H	101.656157400000	97.807811300000	97.691775220000

262	H	103.297124200000	94.453356650000	92.729343980000
263	H	104.092723300000	92.813605120000	95.215107830000
264	H	105.237596300000	93.765324030000	94.249881250000
265	H	103.742643800000	91.288265920000	93.259911470000
266	H	106.024209500000	92.899761010000	91.997268740000
267	H	104.311245100000	92.807217200000	91.425270210000
268	H	105.360388500000	91.360632070000	91.365667540000
269	H	106.643331600000	91.633553630000	94.254812050000
270	H	105.970034200000	90.153075400000	93.486723020000
271	H	105.343142500000	90.711099830000	95.081941440000
272	H	100.116176600000	91.478221720000	94.196422920000
273	H	99.087889360000	90.944905310000	96.466242710000
274	H	99.921743690000	92.380696210000	97.163807830000
275	H	100.866892900000	88.710132140000	95.860438460000
276	H	104.238002400000	90.435714880000	97.831619180000
277	H	96.632779270000	93.921950220000	93.312816980000
278	H	95.301545480000	94.737682920000	95.129891860000
279	H	95.457670390000	91.181980300000	93.969862940000
280	H	95.888097010000	91.832426350000	92.358182650000
281	H	93.965689010000	94.462741700000	92.074287260000
282	H	90.843734920000	92.119264090000	93.681057560000
283	H	99.564948340000	82.227690420000	92.480712420000
284	H	101.146256000000	83.832692260000	93.185369550000
285	H	100.028369000000	85.717159880000	91.040886470000
286	H	101.415304500000	84.609916890000	90.782761140000
287	H	103.450808900000	84.916746430000	92.561487070000
288	H	100.283078200000	87.773748380000	92.059913340000
289	H	104.831525200000	86.629419070000	93.675270400000
290	H	101.669790000000	89.522793050000	93.178767840000
291	H	103.968680900000	88.947074370000	93.995832350000
292	H	97.855234080000	85.176715380000	95.716192740000
293	H	97.204950670000	87.478043750000	95.387087420000
294	H	100.265680300000	86.661679590000	96.921263180000
295	H	99.876839800000	84.925974130000	97.222290050000
296	H	97.906150160000	84.273454110000	98.561646980000
297	H	98.943003380000	88.409772630000	98.009531240000
298	H	96.420783210000	84.926636220000	100.450647800000
299	H	97.414584630000	89.076142690000	99.813479950000
300	H	96.120250920000	87.320603150000	101.061184600000
301	H	99.093670910000	104.604284700000	98.491846930000
302	H	99.949306150000	102.849294000000	99.730944040000
303	H	101.825746900000	101.973760000000	98.472002820000
304	H	98.585531420000	100.882692500000	97.812519610000
305	H	97.692228040000	101.823919700000	99.050614160000

306	H	98.116041970000	99.811921680000	100.190687800000
307	H	99.470264710000	100.826229900000	100.750504100000
308	H	103.927460200000	100.263807900000	97.378827570000
309	H	103.827867700000	97.742777950000	98.607601720000
310	H	104.636454600000	98.805708670000	99.800202530000
311	H	107.983416900000	98.782962730000	96.884380270000
312	H	110.172610300000	98.675653550000	98.384777500000
313	H	109.120081400000	98.263270830000	99.794213000000
314	H	108.976757000000	99.893000980000	99.021929100000
315	H	108.840391000000	94.270231780000	97.310394060000
316	H	107.085133900000	93.191250560000	98.621487330000
317	H	106.980314400000	92.509790880000	96.974058670000
318	H	107.689647700000	94.689141170000	92.891997030000
319	H	110.165765800000	96.097071240000	93.873713890000
320	H	107.574579800000	96.925076780000	91.718687160000
321	H	105.513093400000	96.660756090000	94.029873500000
322	H	105.327682800000	95.885951560000	92.439774080000
323	H	105.292696400000	97.664898630000	92.542029830000
324	H	111.695052700000	87.626336150000	96.728779520000
325	H	109.606435800000	86.735218250000	95.937200690000
326	H	110.920445100000	88.486990810000	93.856346330000
327	H	107.801170300000	88.453184630000	95.215695570000
328	H	108.744091800000	89.654936210000	96.125940990000
329	H	108.555189100000	88.260292580000	98.180236040000
330	H	107.728387500000	86.977013060000	97.245142030000
331	H	106.633339300000	87.580355400000	99.679340420000
332	H	105.153890400000	87.107621680000	98.898409760000
333	H	105.024526700000	88.662479330000	99.704056530000
334	H	103.901029500000	99.744596100000	101.882796300000
335	H	102.270672700000	98.082799970000	101.417657400000
336	H	102.010749700000	97.823372780000	103.167241600000
337	H	104.587425700000	94.408001060000	100.422618300000
338	H	104.889396300000	94.533233380000	102.207866800000
339	H	108.825554400000	96.110458470000	101.337092900000
340	H	109.044225900000	95.062154220000	99.885506670000
341	H	110.503780000000	92.271854880000	103.079295400000
342	H	112.668096100000	92.823351130000	101.234617800000
343	H	110.435969100000	89.942384880000	102.359678200000
344	H	110.649325200000	90.365490480000	100.658390500000
345	H	108.247517100000	92.730024820000	101.464662000000
346	H	108.607366700000	88.484456150000	101.477944900000
347	H	105.865345200000	92.557594180000	100.624176000000
348	H	106.204161800000	88.393684500000	101.251942700000
349	H	102.103423900000	99.255710980000	95.022009960000

350 H	99.618533610000	82.129524800000	100.182487200000
351 H	104.163915000000	83.893485180000	101.657348000000
352 H	96.506447490000	99.086557120000	94.186951710000

Configuration 2

1 N	96.616000000000	96.548000000000	103.238000000000
2 C	95.486000000000	96.561000000000	102.283000000000
3 C	94.928000000000	95.190000000000	102.054000000000
4 O	93.915000000000	95.090000000000	101.318000000000
5 C	95.889000000000	97.269000000000	100.974000000000
6 C	97.009000000000	96.521000000000	100.275000000000
7 N	96.741000000000	95.596000000000	99.274000000000
8 C	98.325000000000	96.811000000000	100.171000000000
9 C	97.852000000000	95.355000000000	98.618000000000
10 N	98.782000000000	96.207000000000	99.020000000000
11 H	97.544770120000	96.356026810000	102.848610000000
12 H	95.821649420000	95.154978570000	99.106505880000
13 N	95.282000000000	94.174000000000	102.811000000000
14 C	94.766000000000	92.822000000000	102.457000000000
15 C	93.637000000000	92.444000000000	103.378000000000
16 O	92.603000000000	91.977000000000	102.930000000000
17 C	95.908000000000	91.797000000000	102.472000000000
18 C	96.851000000000	91.931000000000	101.276000000000
19 N	96.466000000000	91.620000000000	99.994000000000
20 C	98.196000000000	91.891000000000	101.291000000000
21 C	97.540000000000	91.486000000000	99.246000000000
22 N	98.615000000000	91.525000000000	100.031000000000
23 H	96.129689140000	94.295772790000	103.393329900000
24 H	95.492528070000	91.588604620000	99.666859640000
25 N	98.804000000000	91.714000000000	106.949000000000
26 C	97.842000000000	92.804000000000	107.147000000000
27 C	96.796000000000	92.343000000000	108.173000000000
28 O	96.194000000000	91.293000000000	108.112000000000
29 C	97.128000000000	93.149000000000	105.832000000000
30 S	98.138000000000	93.811000000000	104.504000000000
31 H	98.373038170000	90.779136320000	106.912742600000
32 H	99.161041430000	92.863635500000	104.780533700000
33 N	101.175000000000	98.599000000000	94.678000000000
34 C	101.797000000000	97.458000000000	95.392000000000
35 C	101.724000000000	96.240000000000	94.440000000000
36 O	100.854000000000	96.140000000000	93.576000000000
37 C	101.031000000000	97.189000000000	96.689000000000
38 C	101.599000000000	96.057000000000	97.516000000000
39 O	102.197000000000	96.320000000000	98.557000000000
40 O	101.407000000000	94.891000000000	97.101000000000

41 H	101.226785900000	99.472606400000	95.221447860000
42 N	102.618000000000	95.279000000000	94.650000000000
43 C	102.625000000000	94.067000000000	93.820000000000
44 C	101.328000000000	93.311000000000	93.845000000000
45 O	100.989000000000	92.618000000000	92.880000000000
46 C	103.827000000000	93.188000000000	94.183000000000
47 C	104.303000000000	92.255000000000	93.069000000000
48 C	105.832000000000	92.178000000000	92.960000000000
49 C	103.906000000000	90.808000000000	93.365000000000
50 H	103.309709600000	95.332183210000	95.431636300000
51 N	100.577000000000	93.408000000000	94.965000000000
52 C	99.466000000000	92.526000000000	95.197000000000
53 C	98.141000000000	93.274000000000	94.980000000000
54 O	98.102000000000	94.516000000000	94.965000000000
55 C	99.530000000000	91.940000000000	96.631000000000
56 C	100.784000000000	91.129000000000	96.838000000000
57 N	101.895000000000	91.663000000000	97.479000000000
58 C	100.894000000000	89.774000000000	96.994000000000
59 C	102.798000000000	90.698000000000	97.556000000000
60 N	102.249000000000	89.539000000000	97.195000000000
61 H	100.858801300000	94.073609090000	95.733622480000
62 H	102.699409500000	88.617244930000	97.252457610000
63 N	97.015000000000	92.593000000000	95.187000000000
64 C	95.734000000000	93.262000000000	94.858000000000
65 C	95.297000000000	94.202000000000	96.014000000000
66 O	95.255000000000	93.759000000000	97.171000000000
67 C	94.623000000000	92.200000000000	94.717000000000
68 C	93.579000000000	92.453000000000	93.671000000000
69 N	93.521000000000	91.772000000000	92.480000000000
70 C	92.413000000000	93.158000000000	93.744000000000
71 C	92.520000000000	92.255000000000	91.742000000000
72 N	91.861000000000	93.137000000000	92.484000000000
73 H	97.000888470000	91.566650110000	95.344077840000
74 H	94.219299110000	91.085652500000	92.173842910000
75 N	99.411000000000	87.469000000000	94.843000000000
76 C	98.730000000000	88.770000000000	94.614000000000
77 C	97.263000000000	88.696000000000	94.891000000000
78 O	96.698000000000	89.474000000000	95.663000000000
79 C	99.094000000000	89.151000000000	93.179000000000
80 C	100.497000000000	88.623000000000	93.124000000000
81 C	100.421000000000	87.241000000000	93.768000000000
82 N	103.989000000000	100.458000000000	96.844000000000
83 C	104.404000000000	99.164000000000	97.424000000000
84 C	105.865000000000	98.822000000000	97.025000000000

85	O	106.046000000000	98.288000000000	95.905000000000
86	H	104.135516800000	100.539466500000	95.832195980000
87	N	106.659000000000	98.584000000000	98.044000000000
88	C	108.075000000000	98.315000000000	97.818000000000
89	C	108.392000000000	96.805000000000	97.745000000000
90	O	109.494000000000	96.442000000000	97.342000000000
91	C	108.893000000000	98.929000000000	98.959000000000
92	H	106.330567200000	98.633601150000	99.030497150000
93	N	107.294000000000	96.036000000000	97.617000000000
94	C	107.477000000000	94.644000000000	97.180000000000
95	C	107.593000000000	94.531000000000	95.694000000000
96	O	107.965000000000	93.512000000000	95.102000000000
97	C	106.415000000000	93.726000000000	97.778000000000
98	C	104.996000000000	94.229000000000	97.723000000000
99	O	104.663000000000	95.114000000000	96.942000000000
100	O	104.129000000000	93.497000000000	98.285000000000
101	H	106.340867500000	96.388335610000	97.775838620000
102	N	107.385000000000	95.651000000000	94.971000000000
103	C	107.742000000000	95.657000000000	93.536000000000
104	C	109.232000000000	95.667000000000	93.292000000000
105	O	109.647000000000	95.685000000000	92.111000000000
106	C	107.083000000000	96.884000000000	92.865000000000
107	O	107.605000000000	98.065000000000	93.454000000000
108	C	105.566000000000	96.881000000000	93.042000000000
109	H	107.075814000000	96.530271260000	95.421251370000
110	H	107.077193700000	98.323290760000	94.276002540000
111	N	109.143000000000	88.107000000000	96.231000000000
112	C	108.380000000000	87.811000000000	95.016000000000
113	C	109.250000000000	88.123000000000	93.777000000000
114	O	109.039000000000	87.518000000000	92.728000000000
115	C	107.098000000000	88.641000000000	95.016000000000
116	C	106.097000000000	88.251000000000	96.103000000000
117	S	105.356000000000	86.646000000000	95.724000000000
118	C	104.062000000000	87.186000000000	94.656000000000
119	H	109.336695500000	89.087960730000	96.443510940000
120	N	102.103000000000	99.744000000000	102.789000000000
121	C	101.578000000000	98.386000000000	102.490000000000
122	C	102.713000000000	97.427000000000	102.399000000000
123	O	103.195000000000	96.948000000000	103.418000000000
124	H	102.688000700000	99.857447670000	103.615417500000
125	N	103.098000000000	96.960000000000	101.190000000000
126	C	104.181000000000	95.981000000000	101.077000000000
127	C	105.533000000000	96.625000000000	101.294000000000
128	O	105.899000000000	97.580000000000	100.568000000000

129	H	102.637979500000	97.199886060000	100.294363300000
130	N	106.433000000000	95.920000000000	101.953000000000
131	C	107.867000000000	96.307000000000	101.871000000000
132	C	108.682000000000	95.615000000000	102.997000000000
133	O	108.527000000000	95.944000000000	104.181000000000
134	H	106.158932200000	95.191351450000	102.618053800000
135	N	109.348000000000	94.510000000000	102.640000000000
136	C	109.750000000000	93.542000000000	103.613000000000
137	C	111.160000000000	93.027000000000	103.470000000000
138	O	111.688000000000	92.517000000000	104.474000000000
139	C	108.734000000000	92.432000000000	103.867000000000
140	C	107.825000000000	92.056000000000	102.728000000000
141	C	108.191000000000	91.068000000000	101.816000000000
142	C	106.598000000000	92.679000000000	102.536000000000
143	C	107.425000000000	90.781000000000	100.684000000000
144	C	105.722000000000	92.267000000000	101.517000000000
145	C	106.180000000000	91.382000000000	100.543000000000
146	O	105.377000000000	90.994000000000	99.500000000000
147	H	109.340040200000	94.228326420000	101.651130400000
148	H	104.753924400000	91.770194370000	99.268213650000
149	Zn	102.145000000000	93.524000000000	98.419000000000
150	K	98.346000000000	96.625000000000	93.430000000000
151	H	103.920051500000	85.015120090000	101.385536800000
152	O	100.491000000000	93.784000000000	99.561000000000
153	H	99.866947200000	92.991041860000	99.723402660000
154	H	100.015240800000	94.673835140000	99.407285930000
155	O	102.786000000000	92.563000000000	100.449000000000
156	C	102.036000000000	92.429000000000	101.440000000000
157	C	101.852000000000	93.524000000000	102.463000000000
158	N	101.349000000000	91.281000000000	101.633000000000
159	H	100.833897700000	91.140217140000	102.507579300000
160	C	101.410000000000	90.164000000000	100.687000000000
161	C	100.653000000000	88.953000000000	101.230000000000
162	C	101.056000000000	87.720000000000	100.436000000000
163	C	100.314000000000	86.469000000000	100.891000000000
164	C	100.735000000000	85.245000000000	100.095000000000
165	N	100.186000000000	84.033000000000	100.528000000000
166	H	100.280193400000	83.919444900000	101.554231200000
167	C	102.277000000000	84.937000000000	100.223000000000
168	O	102.875000000000	84.915000000000	99.170000000000
169	H	102.771549900000	93.659424370000	103.062864500000
170	H	100.575743400000	85.352664170000	99.008115600000
171	H	101.661238800000	94.468063660000	101.919626000000
172	H	101.014756300000	93.335536620000	103.156805300000

173 H	102.478059700000	89.913806180000	100.518078000000
174 H	100.954318900000	90.484187260000	99.726443830000
175 H	99.561731170000	89.136149300000	101.152081900000
176 C	99.280000000000	83.194000000000	100.000000000000
177 O	99.332000000000	83.112000000000	98.792000000000
178 N	102.899000000000	85.132000000000	101.382000000000
179 H	100.914989800000	88.794827580000	102.297734900000
180 H	100.843583400000	87.894091040000	99.359612050000
181 H	102.148732700000	87.553123500000	100.535060900000
182 H	99.218341830000	86.607584510000	100.774253800000
183 H	100.497981300000	86.302162320000	101.975017800000
184 H	96.588808860000	97.186264560000	104.034721300000
185 H	94.654580410000	97.123784660000	102.751202600000
186 H	96.226288440000	98.289081420000	101.220994600000
187 H	95.014297270000	97.329884550000	100.299504600000
188 H	98.910074020000	97.555665800000	100.706563600000
189 H	97.918000310000	94.719140050000	97.734430620000
190 H	94.301551710000	92.926955540000	101.455591000000
191 H	93.783543150000	92.640117040000	104.468062500000
192 H	95.481525490000	90.772832110000	102.466671100000
193 H	96.514708230000	91.912681910000	103.387555100000
194 H	98.870958340000	92.057166830000	102.123550800000
195 H	97.510824730000	91.237435930000	98.190851080000
196 H	99.675569870000	91.765052530000	107.485546700000
197 H	98.371618620000	93.694775970000	107.528175100000
198 H	96.606467820000	93.045279140000	109.021141600000
199 H	96.350268820000	93.909269170000	106.041726100000
200 H	96.596601030000	92.226865730000	105.518848000000
201 H	101.521464000000	98.706685870000	93.715013170000
202 H	102.871755400000	97.618767640000	95.632983050000
203 H	99.979117050000	96.955946680000	96.438094670000
204 H	101.047640900000	98.101829940000	97.309886900000
205 H	102.736581400000	94.390928940000	92.764545150000
206 H	103.600532300000	92.612244450000	95.107280420000
207 H	104.655417100000	93.884717670000	94.441784390000
208 H	103.860444300000	92.563172970000	92.100965360000
209 H	106.277444900000	93.131860440000	92.614117810000
210 H	106.135462800000	91.391091680000	92.245292320000
211 H	106.294183600000	91.968129310000	93.945088710000
212 H	104.210045700000	90.134596680000	92.541968030000
213 H	102.808167000000	90.708130470000	93.500312600000
214 H	104.414082900000	90.471686790000	94.292878680000
215 H	99.551606160000	91.710020180000	94.446295640000
216 H	98.660782920000	91.277922440000	96.781884750000

217	H	99.490453290000	92.765269040000	97.369701830000
218	H	100.264775900000	88.944284870000	96.653160060000
219	H	103.820298300000	90.791464090000	97.917517570000
220	H	95.852501560000	93.834503810000	93.920921180000
221	H	94.973640930000	95.235039060000	95.768878190000
222	H	94.133421410000	92.061974550000	95.701263600000
223	H	95.106520180000	91.222211720000	94.506694130000
224	H	92.017171530000	93.784405440000	94.540650990000
225	H	92.281851040000	91.960050950000	90.723179170000
226	H	98.938690250000	86.692292030000	95.315147800000
227	H	99.123795440000	89.568836310000	95.279674480000
228	H	96.701254010000	87.865512730000	94.400397940000
229	H	99.041852640000	90.237801480000	92.985254540000
230	H	98.444655330000	88.612294790000	92.458730030000
231	H	101.156388700000	89.277970550000	93.728147620000
232	H	100.911210900000	88.594025710000	92.101282060000
233	H	100.082344700000	86.480093000000	93.035626810000
234	H	101.387162700000	86.925009330000	94.197006480000
235	H	104.239291700000	101.292140500000	97.381800570000
236	H	103.781785600000	98.337222490000	97.040527370000
237	H	104.288162000000	99.203561800000	98.522501510000
238	H	108.366702100000	98.717912820000	96.830515820000
239	H	109.949196600000	98.627452910000	98.848458120000
240	H	108.508469800000	98.557663230000	99.927035580000
241	H	108.827967200000	100.030805200000	98.946038220000
242	H	108.475281900000	94.327351140000	97.545174980000
243	H	106.676435000000	93.487181770000	98.829886730000
244	H	106.443415500000	92.764318340000	97.223764700000
245	H	107.352911600000	94.725546710000	93.076902470000
246	H	109.908544200000	95.675111990000	94.174759640000
247	H	107.392421700000	96.881725450000	91.805827490000
248	H	105.299293200000	96.930586310000	94.114240410000
249	H	105.103238200000	95.974497050000	92.604558930000
250	H	105.151906100000	97.769734820000	92.532704090000
251	H	109.810020300000	87.415632280000	96.575489900000
252	H	108.119721700000	86.734843690000	94.974819190000
253	H	110.070918700000	88.869120370000	93.911116440000
254	H	106.625880900000	88.557206980000	94.014400870000
255	H	107.378619500000	89.711656160000	95.136572140000
256	H	105.313769800000	89.027639710000	96.205879540000
257	H	106.639998800000	88.159337830000	97.062266390000
258	H	104.431062200000	87.734659540000	93.767852120000
259	H	103.318809500000	87.836873960000	95.159863570000
260	H	103.535482600000	86.289905680000	94.285667850000

261 H	101.763479500000	100.558412000000	102.285825700000
262 H	101.030713500000	98.447941580000	101.530984100000
263 H	100.892019800000	97.999526840000	103.275367700000
264 H	104.154651700000	95.579610930000	100.046614900000
265 H	104.014416300000	95.157994600000	101.798334100000
266 H	107.935038600000	97.398562730000	102.025942300000
267 H	108.243673800000	96.071481680000	100.855267600000
268 H	109.801547400000	94.129191670000	104.558746600000
269 H	111.711372900000	93.162600470000	102.512055700000
270 H	108.093765600000	92.756762400000	104.710419400000
271 H	109.282706900000	91.543412180000	104.235991000000
272 H	109.140101000000	90.535388760000	101.958901200000
273 H	106.242080200000	93.379839040000	103.304053800000
274 H	107.742486900000	90.036430980000	99.947265680000
275 H	104.713677300000	92.688599440000	101.413724700000
276 H	98.684962210000	82.564941670000	100.692765500000
277 H	102.423258000000	85.190132490000	102.285728700000

Configuration 3

1 N	96.619000000000	96.292000000000	103.369000000000
2 C	95.544000000000	96.353000000000	102.362000000000
3 C	94.937000000000	95.020000000000	102.029000000000
4 O	93.939000000000	94.971000000000	101.270000000000
5 C	96.051000000000	97.046000000000	101.089000000000
6 C	97.144000000000	96.313000000000	100.336000000000
7 N	96.851000000000	95.419000000000	99.323000000000
8 C	98.431000000000	96.671000000000	100.162000000000
9 C	97.955000000000	95.178000000000	98.651000000000
10 N	98.889000000000	96.024000000000	99.057000000000
11 H	97.567985530000	96.178659790000	102.994351900000
12 H	95.933734880000	94.977547530000	99.152220950000
13 N	95.374000000000	93.921000000000	102.682000000000
14 C	94.791000000000	92.621000000000	102.380000000000
15 C	93.610000000000	92.319000000000	103.284000000000
16 O	92.657000000000	91.696000000000	102.838000000000
17 C	95.850000000000	91.528000000000	102.426000000000
18 C	96.829000000000	91.492000000000	101.263000000000
19 N	96.451000000000	91.565000000000	99.951000000000
20 C	98.184000000000	91.516000000000	101.276000000000
21 C	97.543000000000	91.559000000000	99.197000000000
22 N	98.593000000000	91.461000000000	99.976000000000
23 H	96.243377200000	94.019529230000	103.236461500000
24 H	95.488350840000	91.637320630000	99.601777210000
25 N	98.782000000000	91.681000000000	106.921000000000
26 C	97.867000000000	92.804000000000	107.037000000000

27 C	96.790000000000	92.456000000000	108.102000000000
28 O	96.301000000000	91.306000000000	108.026000000000
29 C	97.186000000000	93.079000000000	105.685000000000
30 S	98.224000000000	93.829000000000	104.443000000000
31 H	98.307034080000	90.767259740000	106.954820000000
32 H	99.223809580000	92.837626880000	104.604894000000
33 N	101.160000000000	98.438000000000	94.894000000000
34 C	101.880000000000	97.345000000000	95.581000000000
35 C	101.859000000000	96.127000000000	94.669000000000
36 O	101.016000000000	96.042000000000	93.765000000000
37 C	101.129000000000	97.052000000000	96.896000000000
38 C	101.831000000000	95.999000000000	97.723000000000
39 O	102.689000000000	96.304000000000	98.544000000000
40 O	101.520000000000	94.803000000000	97.455000000000
41 H	100.299610400000	98.056283990000	94.463176960000
42 N	102.805000000000	95.212000000000	94.873000000000
43 C	102.817000000000	94.003000000000	94.040000000000
44 C	101.532000000000	93.170000000000	94.208000000000
45 O	101.236000000000	92.361000000000	93.314000000000
46 C	104.037000000000	93.140000000000	94.427000000000
47 C	104.492000000000	92.154000000000	93.381000000000
48 C	105.991000000000	92.053000000000	93.173000000000
49 C	104.080000000000	90.729000000000	93.695000000000
50 H	103.468182100000	95.255584310000	95.677900860000
51 N	100.793000000000	93.359000000000	95.276000000000
52 C	99.622000000000	92.465000000000	95.483000000000
53 C	98.315000000000	93.243000000000	95.322000000000
54 O	98.282000000000	94.492000000000	95.276000000000
55 C	99.652000000000	91.891000000000	96.899000000000
56 C	100.870000000000	91.061000000000	97.177000000000
57 N	101.996000000000	91.562000000000	97.812000000000
58 C	101.016000000000	89.722000000000	97.180000000000
59 C	102.917000000000	90.598000000000	97.876000000000
60 N	102.301000000000	89.450000000000	97.559000000000
61 H	101.015543100000	94.071512720000	96.020984440000
62 H	102.740116400000	88.520083610000	97.575818380000
63 N	97.241000000000	92.493000000000	95.190000000000
64 C	95.917000000000	93.076000000000	94.949000000000
65 C	95.428000000000	93.906000000000	96.100000000000
66 O	95.563000000000	93.460000000000	97.244000000000
67 C	94.864000000000	91.980000000000	94.662000000000
68 C	93.832000000000	92.352000000000	93.600000000000
69 N	93.512000000000	91.507000000000	92.572000000000
70 C	92.749000000000	93.164000000000	93.784000000000

71 C	92.352000000000	91.864000000000	92.072000000000
72 N	91.904000000000	92.938000000000	92.725000000000
73 H	97.304128340000	91.457682610000	95.247926590000
74 H	94.114523270000	90.745472850000	92.243259090000
75 N	99.835000000000	87.143000000000	94.376000000000
76 C	99.396000000000	88.519000000000	94.577000000000
77 C	97.897000000000	88.687000000000	94.611000000000
78 O	97.321000000000	89.505000000000	95.358000000000
79 C	100.101000000000	89.355000000000	93.503000000000
80 C	101.242000000000	88.498000000000	93.079000000000
81 C	100.784000000000	87.067000000000	93.231000000000
82 N	104.376000000000	100.684000000000	96.942000000000
83 C	104.846000000000	99.371000000000	97.363000000000
84 C	106.363000000000	99.384000000000	97.556000000000
85 O	107.065000000000	99.835000000000	96.616000000000
86 H	103.383087100000	100.767097100000	96.710267190000
87 N	106.891000000000	98.694000000000	98.529000000000
88 C	108.279000000000	98.251000000000	98.529000000000
89 C	108.435000000000	96.780000000000	98.279000000000
90 O	109.552000000000	96.381000000000	97.879000000000
91 C	108.997000000000	98.621000000000	99.823000000000
92 H	106.314889200000	98.337237750000	99.312292870000
93 N	107.355000000000	96.033000000000	98.038000000000
94 C	107.529000000000	94.699000000000	97.455000000000
95 C	107.486000000000	94.720000000000	95.938000000000
96 O	107.886000000000	93.723000000000	95.303000000000
97 C	106.558000000000	93.683000000000	98.059000000000
98 C	105.093000000000	94.031000000000	97.931000000000
99 O	104.767000000000	94.974000000000	97.177000000000
100 O	104.239000000000	93.436000000000	98.621000000000
101 H	106.431060900000	96.270978790000	98.428311840000
102 N	107.458000000000	95.941000000000	95.383000000000
103 C	107.748000000000	96.146000000000	93.958000000000
104 C	109.274000000000	96.167000000000	93.735000000000
105 O	109.692000000000	96.313000000000	92.609000000000
106 C	107.156000000000	97.482000000000	93.530000000000
107 O	107.672000000000	98.550000000000	94.312000000000
108 C	105.627000000000	97.516000000000	93.573000000000
109 H	107.270004000000	96.767375520000	95.962455650000
110 H	107.129712600000	98.985622180000	95.047585620000
111 N	109.048000000000	88.895000000000	96.780000000000
112 C	108.224000000000	88.507000000000	95.605000000000
113 C	108.917000000000	88.846000000000	94.305000000000
114 O	108.789000000000	88.150000000000	93.292000000000

115 C	106.833000000000	89.169000000000	95.703000000000
116 C	106.039000000000	88.562000000000	96.863000000000
117 S	105.423000000000	86.932000000000	96.387000000000
118 C	104.199000000000	87.405000000000	95.120000000000
119 H	109.207899000000	89.899355010000	96.908051510000
120 N	102.081000000000	99.677000000000	102.847000000000
121 C	101.770000000000	98.294000000000	102.396000000000
122 C	103.018000000000	97.406000000000	102.502000000000
123 O	103.531000000000	97.250000000000	103.586000000000
124 H	102.464458800000	99.724396520000	103.797801200000
125 N	103.339000000000	96.677000000000	101.440000000000
126 C	104.333000000000	95.633000000000	101.559000000000
127 C	105.734000000000	96.265000000000	101.559000000000
128 O	106.207000000000	96.649000000000	100.507000000000
129 H	102.939171500000	96.808241730000	100.485979800000
130 N	106.427000000000	96.182000000000	102.686000000000
131 C	107.913000000000	96.158000000000	102.637000000000
132 C	108.511000000000	95.227000000000	103.647000000000
133 O	108.228000000000	95.364000000000	104.861000000000
134 H	105.956242300000	96.098742530000	103.598857600000
135 N	109.085000000000	94.098000000000	103.244000000000
136 C	109.540000000000	93.115000000000	104.205000000000
137 C	110.916000000000	92.606000000000	103.915000000000
138 O	111.334000000000	91.507000000000	104.321000000000
139 C	108.490000000000	92.007000000000	104.370000000000
140 C	107.675000000000	91.687000000000	103.140000000000
141 C	108.163000000000	90.857000000000	102.130000000000
142 C	106.451000000000	92.316000000000	102.905000000000
143 C	107.391000000000	90.491000000000	101.038000000000
144 C	105.701000000000	92.023000000000	101.749000000000
145 C	106.125000000000	91.052000000000	100.873000000000
146 O	105.487000000000	90.836000000000	99.667000000000
147 H	109.133518800000	93.858649070000	102.245767300000
148 H	104.968341500000	91.686493840000	99.423862300000
149 Zn	102.258000000000	93.423000000000	98.752000000000
150 H	102.635550600000	89.828525720000	100.978470000000
151 H	101.209673700000	90.472221660000	100.090299800000
152 C	99.506000000000	83.228000000000	99.710000000000
153 O	99.924000000000	83.313000000000	98.575000000000
154 H	99.650055330000	89.182436120000	101.387753800000
155 H	100.883875700000	88.776276670000	102.636194200000
156 H	101.070767500000	87.932825490000	99.701135160000
157 H	102.171942300000	87.460614600000	101.017433900000
158 H	99.205373280000	86.672394200000	100.681604800000

159 H	100.134557900000	86.348311020000	102.171266800000
160 H	101.202199000000	85.416696090000	99.372618440000
161 N	102.051000000000	84.497000000000	102.524000000000
162 H	101.350278700000	84.886685500000	103.160920100000
163 O	100.613000000000	93.710000000000	99.887000000000
164 H	99.963367540000	92.918913350000	99.926660200000
165 H	100.135824700000	94.567835850000	99.580456650000
166 O	102.899000000000	92.538000000000	100.800000000000
167 C	102.155000000000	92.413000000000	101.794000000000
168 C	101.953000000000	93.527000000000	102.798000000000
169 N	101.498000000000	91.254000000000	102.017000000000
170 H	101.012320600000	91.115135940000	102.909459300000
171 C	101.572000000000	90.131000000000	101.083000000000
172 C	100.723000000000	88.950000000000	101.550000000000
173 C	101.114000000000	87.717000000000	100.790000000000
174 C	100.229000000000	86.511000000000	101.077000000000
175 C	100.876000000000	85.263000000000	100.412000000000
176 N	100.146000000000	84.052000000000	100.546000000000
177 H	99.949088530000	83.857790580000	101.543692000000
178 C	102.191000000000	84.885000000000	101.248000000000
179 O	103.125000000000	84.558000000000	100.537000000000
180 H	102.850918200000	93.635847620000	103.434126700000
181 H	101.801742400000	94.464897960000	102.232965000000
182 H	101.079842700000	93.359930640000	103.450890400000
183 H	96.544716710000	96.914741260000	104.176723100000
184 H	94.707481190000	96.951454740000	102.772462000000
185 H	96.433011100000	98.041736660000	101.375149000000
186 H	95.197600840000	97.199158080000	100.399312000000
187 H	99.011707190000	97.430143010000	100.681742300000
188 H	98.008280100000	94.588315340000	97.734947710000
189 H	94.334399370000	92.703305320000	101.370046700000
190 H	93.644145350000	92.721593140000	104.324195600000
191 H	95.325551350000	90.548809780000	102.478751300000
192 H	96.445627000000	91.618111960000	103.354116700000
193 H	98.868116870000	91.517456660000	102.118981300000
194 H	97.496736900000	91.627961430000	98.116304220000
195 H	99.651211280000	91.734069500000	107.461205700000
196 H	98.425790490000	93.708370950000	107.341684400000
197 H	96.460930470000	93.190475590000	108.866944500000
198 H	96.339710580000	93.779836620000	105.831472500000
199 H	96.752889380000	92.114598460000	105.353767300000
200 H	101.739371400000	98.901911660000	94.178708870000
201 H	102.918341700000	97.659261770000	95.804600210000
202 H	100.105338600000	96.710014100000	96.654815210000

203	H	101.073969700000	97.998934580000	97.456088310000
204	H	102.846459200000	94.290080690000	92.972142930000
205	H	103.820720800000	92.627932490000	95.391743720000
206	H	104.868335900000	93.848712400000	94.639976470000
207	H	104.017896300000	92.430950390000	92.414805060000
208	H	106.443494800000	93.011706580000	92.848806510000
209	H	106.231506300000	91.288425400000	92.412818740000
210	H	106.506332500000	91.795440770000	94.119503810000
211	H	104.401743000000	90.030605060000	92.899199570000
212	H	102.977669700000	90.654434580000	93.796854150000
213	H	104.552062300000	90.413196070000	94.649111380000
214	H	99.689296400000	91.686282500000	94.704393480000
215	H	98.780487660000	91.225416410000	97.011088670000
216	H	99.590797220000	92.714495870000	97.635483120000
217	H	102.830297400000	83.965359070000	102.931681500000
218	H	100.328037900000	88.916554310000	96.962266770000
219	H	103.918821700000	90.650487180000	98.294268230000
220	H	96.002147980000	93.726798320000	94.054753170000
221	H	94.907724170000	94.863809220000	95.892531020000
222	H	94.348744890000	91.689299110000	95.597401950000
223	H	95.417574560000	91.078791740000	94.327237940000
224	H	92.568984270000	93.947992620000	94.516155800000
225	H	91.881292650000	91.420751660000	91.198150970000
226	H	99.710374730000	86.402745140000	95.066461880000
227	H	99.720206860000	88.920529160000	95.546204030000
228	H	97.318199550000	87.981723900000	93.967679170000
229	H	100.433802500000	90.357731800000	93.822148670000
230	H	99.413324070000	89.516406610000	92.645082560000
231	H	102.091670400000	88.666000400000	93.770340850000
232	H	101.597879800000	88.738628720000	92.062244220000
233	H	100.273847600000	86.707928060000	92.312226180000
234	H	101.616896100000	86.375328880000	93.455815220000
235	H	105.033540900000	101.187488500000	96.335608060000
236	H	104.690257400000	98.540675340000	96.622474010000
237	H	104.329602700000	99.053983440000	98.290873990000
238	H	108.757850000000	98.750336400000	97.662972170000
239	H	110.019713200000	98.206477800000	99.793935440000
240	H	108.447277400000	98.194444790000	100.682361300000
241	H	109.046515600000	99.717819660000	99.935546420000
242	H	108.571159000000	94.395401960000	97.678335430000
243	H	106.804413200000	93.509749660000	99.127671420000
244	H	106.734054100000	92.718851240000	97.533681160000
245	H	107.327292000000	95.311175300000	93.365142390000
246	H	109.912749600000	96.063508140000	94.642843840000

247 H	107.510456500000	97.650471690000	92.493549180000
248 H	105.282828800000	98.511166530000	93.240839850000
249 H	105.268124400000	97.328004740000	94.605268200000
250 H	105.171364400000	96.744346640000	92.922932560000
251 H	109.830891000000	88.281163670000	97.017817200000
252 H	108.068898400000	87.408015310000	95.618210170000
253 H	109.567199600000	89.756059590000	94.316869320000
254 H	106.302509100000	89.050968600000	94.739373020000
255 H	106.975111500000	90.260188900000	95.858802620000
256 H	105.218854400000	89.234709640000	97.171695550000
257 H	106.720892600000	88.434951420000	97.724802900000
258 H	104.666882500000	87.655347590000	94.152021700000
259 H	103.591001700000	88.272302960000	95.438573210000
260 H	103.538237000000	86.535427990000	94.969370120000
261 H	102.553276200000	100.268882600000	102.157072100000
262 H	101.369343400000	98.335546740000	101.367041400000
263 H	101.009070500000	97.852812320000	103.067193100000
264 H	104.274169400000	94.971812810000	100.673500600000
265 H	104.164310100000	95.062414730000	102.489057100000
266 H	108.318086700000	97.169156230000	102.841675000000
267 H	108.180687900000	95.906860890000	101.592693100000
268 H	109.601262000000	93.676777740000	105.170280700000
269 H	111.583145900000	93.303676720000	103.353778900000
270 H	107.792723200000	92.314510180000	105.171503900000
271 H	109.024138600000	91.102556850000	104.726479500000
272 H	109.159731400000	90.409497140000	102.247077300000
273 H	106.074954900000	93.054957610000	103.624925900000
274 H	107.772479800000	89.815452280000	100.264553900000
275 H	104.755728200000	92.541591610000	101.546562300000
276 H	98.771176740000	82.511287960000	100.127792000000
